# Morphology and distribution of sensilla on the antennae and mouthparts of adult *Monochamus saltuarius* Gebler (Coleoptera: Cerambycidae)

**DOI:** 10.3389/finsc.2025.1675406

**Published:** 2025-10-21

**Authors:** Jianjun Wang, Jianguo Wang, Jingxian Wang, Xu Jiang, Shitan Ren, Chuanwang Cao

**Affiliations:** ^1^ Liaoning Provincial Key Laboratory of Forest Protection, Liaoning Academy of Forestry Sciences, Shenyang, China; ^2^ Key Laboratory of Sustainable Forest Ecosystem Management-Ministry of Education, Northeast Forestry University, Harbin, China

**Keywords:** antennae, maxillary and labial palps, sensilla, *Monochamus saltuarius* Gebler, scanning electron microscopy (SEM), chemoreceptors

## Abstract

*Monochamus saltuarius* is an important wood-boring pest of forests and a vector insect for the transmission of *Bursaphelenchus xylophilus* in China and other East Asian regions. To gain insight into the *Mo. saltuarius* olfactory system, we characterized the sizes and morphological characteristics of sensilla on antennae, maxillary palps, and labial palps of adults by scanning electron microscopy. Eight types of antennal sensilla were identified on the antennae: Böhm bristles (BBs), sensilla chaetica (SChs, with subtypes SChI and SChII), sensilla trichodea (STs, with subtypes STI, STII and STIII), sensilla auricillica (SAus), sensilla basiconica (SBs, with subtypes SBI and SBII), sensilla grooved peg (SGPs), dome shaped organs (DSOs), and cuticular pores (CPs); among these, BBs, STIs, STIIs, SChIs, and SChIIs may be mechanoreceptors, and STIIIs, SAus, SBIs, SBIIs, SGPs and CPs may be chemoreceptors. Seven sensillum types were identified on maxillary palps and labial palps: BBs, STs (with subtypes STII, and STIII), SChs, sensilla placodea (SPs), sensilla coeloconica (SCos), CPs, and sensilla twig basiconica (STBs, with subtypes STBI, STBII, STBIII, and STBIV), among which BBs, STIIs, and SChs may be mechanoreceptors, and STIIIs, SPs, CPs, STBIs, STBIIs, STBIIIs, and STIVs may be chemoreceptors. DSOs on the antennae and SCos on the palps may be hydroreceptors, and/or thermoreceptors. The types and densities of sensilla increased from the base to the tip of the antennae, and sensilla with chemical-sensing functions were concentrated mostly on the flagellum. Identification of these sensillum types provides a basis for analyzing the mechanisms of host recognition and environmental perception of *Mo. saltuarius*.

## 1 Introduction

Antennae, maxillary palps, and labial palps are the major organs with which insects perceive external stimuli, and their surfaces are covered with many different types of sensillum. These sensilla enable insects to perceive the external environment and carry out chemical communication, playing an important role in the acquisition of food, detection of host plants, avoidance of predators, identification of mates, and selection of oviposition sites (1–3). The sensillum is a specialized exoskeletal region that comprises formative cells, sensory neurons (which detect stimuli from the outer cuticular structure), and occasionally auxiliary cells (4). Specific sensilla respond to infrared radiation, CO_2_, chemical and mechanical stimuli, temperature, and humidity (5). They function as discrete sensory units and include a variety of types, each with distinct morphological characteristics. Sensilla often exhibit sexual dimorphism within a species, and antennae may be twice as long in males as in females, potentially as a result of male competition (6) or to enable the contact detection of female sex pheromones (7). The antennae, maxillary palps, and labial palps vary substantially in length and morphology; they function in the recognition of potential mates, host plants, and conspecifics and also help to mediate feeding and egg-laying behaviors (1, 3, 7, 8). Because evolutionary forces have shaped antennal architecture to optimize the perception of chemosensory signals (3), detailed structural characterization of antennae, maxillary palps, and labial palps is critical for understanding the chemical ecology of insects, including longhorned beetles.

The long-horned beetle *Monochamus saltuarius* Gebler (Coleoptera: Cerambycidae) is found in the Heilongjiang, Jilin, Liaoning, Inner Mongolia, Gansu, Xinjiang, Hebei, Shanxi, Shandong, and Zhejiang provinces of China (9–12). It is also present in Europe, Russia (Siberia and Sakhalin), Mongolia, South Korea, and Japan. In China, the predicted suitable habitats of *Mo. saltuarius* are mainly located north of 33° latitude (13). Its host species include *Picea asperata*, *Pinus koraiensis*, *Pinus sylvestris* var. *mongholica*, *Larix* spp., *Abies fabri*, and *Pinus tabulaeformis* (9, 10). The larvae of *Mo. saltuarius* penetrate into the host xylem and cut worm channels, thereby reducing wood value (14). The adults of *Mo. saltuarius* gnaw on the bark of branches, affecting the growth of standing trees. More importantly, *Mo. saltuarius* is a vector insect for the transmission of pine wood nematodes (*Bursaphelenchus xylophilus*) in Liaoning (11, 12), Japan (15, 16), South Korea (17, 18), and other East Asian regions. The maximum *B. xylophilus* carrier rate of *Mo. saltuarius* to pine wood was 95%, the maximum carrier number was 9528 (15), and the average carrier number was 7970 (12). Pine wood nematodes cause large numbers of pine deaths in these areas, leading to serious economic losses and ecological damage (12). Huh et al. found that both *Mo. alternatus* and *Mo. saltuarius* have four types (comprising nine subtypes) of antennal sensilla (19). However, there are no reports describing the types and distributions of sensilla on the maxillary and labial palps of adult *Mo. saltuarius*. In this study, we used scanning electron microscopy to observe the morphology and structural composition of antennae, maxillary palps, and labial palps, as well as the types, numbers, and distributions of sensilla in adult *Mo. saltuarius*. Differences were identified in these organs and sensilla between males and females, providing a foundation for future work on the internal structures, sensory mechanisms, and host recognition of various sensilla.

## 2 Materials and methods

### 2.1 Insects

Adults of *Mo. saltuarius* were collected from Hexi Village, Shangjiahe Town, Xinbin Manchu Autonomous County, Liaoning Province (41.85°N–41.86°N, 124.37°E–124.38°E) using hanging traps in the forest. The traps were made by Beijing Zhongjie Sifang Biotechnology (Model L002) and contained mainly pinene, L-β-pinene, and (1S)-(+)-3-carene as attractants.

### 2.2 Sample preparation for scanning electron microscopy

From January to August 2022, the antennae, maxillary palps, and labial palps of male and female *Mo. saltuarius* were removed under a stereomicroscope (Leica M205A) using a scalpel and forceps, then placed separately into 10.0-mL sterilized centrifuge tubes. The samples were fixed in 2.5% glutaraldehyde for 12 hours at 4°C and then ultrasonicated twice for 50 seconds each in 0.1 M phosphate buffered saline (PBS). The samples were dehydrated in a graded ethanol series (15 minutes each at 30%, 50%, 70%, 85%, and 100%), dried twice for 10 minutes each in 100% acetone, and finally air dried for 24 hours. The samples were affixed to copper stubs using double-sided adhesive tape in both ventral and dorsal orientations. An ion sputtering instrument (model KYKY SBC-12, KYKY Technology) was used to deposit an approximately 20-nm conductive layer of gold. The samples were observed, photographed, and measured using a scanning electron microscope (Thermo Fisher Scientific Apreo C) at 20 kV. Forty body length, six antennae (three dorsal surfaces and three ventral surfaces), six maxillary palps (three dorsal surfaces and three ventral surfaces), and six labial palps (three dorsal surfaces and three ventral surfaces) were observed for males and females.

### 2.3 Sensillum identification

The sensillum types of the antennae, maxillary palps, and labial palps were classified mainly according to the sensilla naming system established by Dyer and Seabrook (20), Schneider (4), Altner and Prillinger (21), and Zacharuk (22). We also consulted recent literature on the classification of antennae, maxillary palps, and labial palps in adult Coleoptera (23–27).

### 2.4 Terminology and data analysis

The lengths of each segment of the antennae, maxillary palps, and labial palps, as well as the lengths and basal diameters of the sensilla, were measured using the scale of the SEM. The surface area of each segment was calculated by multiplying the length by the width. Independent sample *t*-tests performed in SPSS (version 25.0, *P*<0.05) were used to assess the significance of differences in sizes and areas of antennae, maxillary palps, and labial palps, as well as lengths, widths, basal diameters, densities, and numbers of various sensilla between males and females. Data were expressed as mean±standard error.

## 3 Results

### 3.1 Morphological structures of antennae and maxillary and labial palps

#### 3.1.1 General antennal morphology

**Figure 1:**
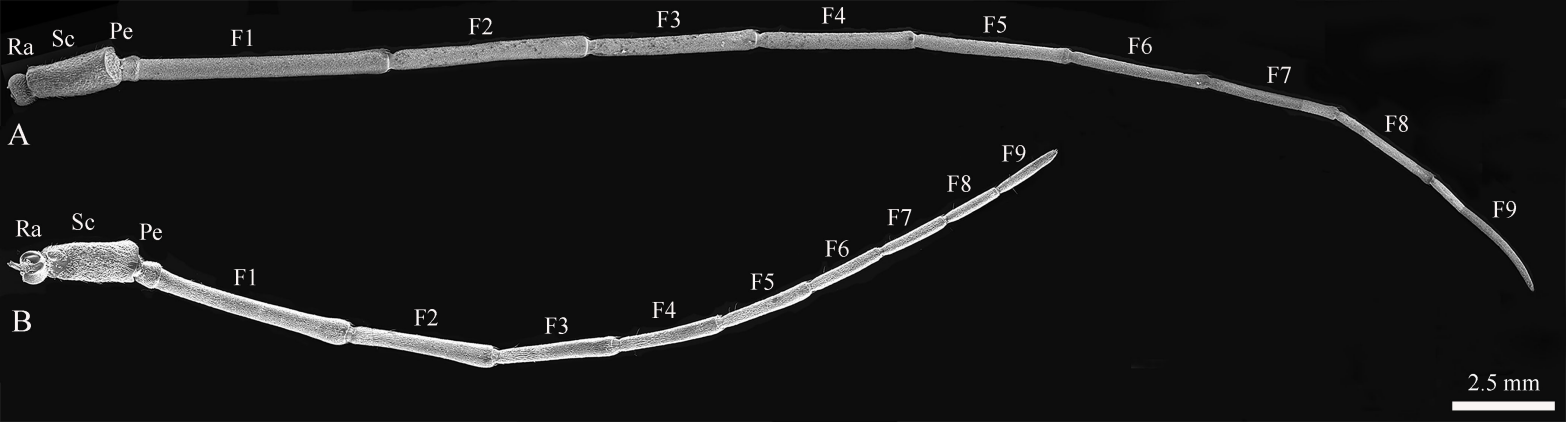
The antennal structures of adult *Mo. saltuarius*. **(A)** Male antennae; **(B)** female antennae. Ra, antennifer/basal radicle; Pe, pedicel; Sc, scape; F1–F9, first to ninth flagellomeres.

The body length of female *Mo. saltuarius* was 19.80±0.20 mm (*n=*40), which was significantly greater (*t*=2.546, *df*=78, *P*=0.013) than that of male *Mo. saltuarius* (19.11±0.20 mm, *n=*40). Metastethidium width did not differ between females and males (5.42±0.12 mm *vs*. 5.41±0.11 mm, *t*=−0.306, *df*=78, *P*=0.760). Like those of most Cerambycidae, the antennae of female and male *Mo. saltuarius* were the same in shape and structure; both were linear and comprised a radicle (Ra), scape (Sc), pedicel (Pe), and long flagellum (F). The flagellum comprised nine flagellomeres, F1–F9 (**Figures 1A, B**). The radicle was connected to the acetabular antennal fossa of the head and face, and the segments were connected by joints. Under an optical microscope, the antennae of male *Mo. saltuariu*s were completely black, whereas those of females were black on the bottom, with black and white chequered flagella.

The total antennal length in females was 26,345.54±363.49 μm; most of this length was accounted for by the flagellum (~87.57%), followed by the scape (~7.88%), the pedicel (~2.46%), and the radicle (~2.09%) (**Table 1**). In female *Mo. saltuarius*, the flagellomeres gradually became shorter from F1 to F8, whereas F9 was longer, about equal to F7 (**Table 1**). The total antennal length in males was 39,675.16±570.12 μm, significantly longer than that of females (*t*=19.71, *df*=10, *P*=0.000). Again, most of the antennal length was accounted for by the flagellum (~91.58%), followed by the scape (~5.94%), the radicle (~1.28%), and the pedicel (~1.19%) (**Table 1**). From F1 to F8, the flagellomeres of males gradually became shorter, whereas F9 was longer, about equal to F6 or F7. In general, each flagellomere was significantly longer in males than in females (**Table 1**), although the antennal pedicel was significantly shorter in males (*t*=−6.150, *df*=10, *P*=0.000).

**Table 1:** Characteristics of antennal segments of female and male *Mo. saltuarius.*.

Antennal segment		Length (μm)		Width (μm)		Surface area (10^5^ μm^2^)	
		♀	♂	♀	♂	♀	♂
Antennifer		550.29±15.11a	508.84±14.02a	680.80±18.04b	846.83±28.54a	3.75±0.17b	4.30±0.12a
Scape		2076.84±64.87b	2357.12±95.60a	908.04±14.86b	1146.85±51.68a	18.83±0.45b	27.22±2.18a
Pedicel		648.21±41.53a	473.07±10.82b	593.77±9.56b	713.70±22.68a	3.85±0.26a	3.38±0.16a
Flagellum	F1	4691.25±61.74b	6834.55±41.62a	481.22±11.90b	622.65±15.56a	22.58±0.69b	42.57±1.18a
	F2	3451.49±94.93b	5103.86± 51.91a	441.71±11.61b	565.54±5.09a	15.26±0.64b	28.87±0.47a
	F3	2941.96±76.41b	4326.83±75.98a	410.40±11.48b	482.44±11.91a	12.08±0.49b	20.88±0.69a
	F4	2568.94±51.74b	3870.77±121.92a	369.61±11.84b	400.10±5.52a	9.49±0.36b	15.52±0.67a
	F5	2339.11±54.37b	3722.07±104.29a	340.35±10.22a	359.94±9.34a	7.96±0.30b	13.42±0.60a
	F6	1904.38±31.75b	3256.85±88.27a	319.37±7.19a	330.84±5.09a	6.07±0.09b	10.77±0.32a
	F7	1809.18±34.93b	3265.83±63.15a	288.13±7.09a	304.25±4.83a	5.21±0.15b	9.92±0.09a
	F8	1550.17±37.50b	2762.02±88.15a	277.04±5.71a	282.84±7.92a	4.29±0.12b	7.79±0.15a
	F9	1813.73±46.38b	3193.37±219.50a	263.97±7.68a	248.34±7.81a	4.80±0.23b	7.87±0.42a
Total		26345.54±363.49b	39675.16±570.12a	—	—	114.17±2.99b	192.50±3.78a

Data are presented as mean±SE. Within a row, different lowercase letters indicate significant differences between the sexes (*t*-test, *P*<0.05). *n*=6 per sex. “—” indicates absence.

In females, the scape was the widest antennal segment (908.04±14.86 μm) and was 3.44 times wider than the narrowest segment, F9; the scape was also the widest segment in males (1146.85±51.68 μm) and was 4.62 times wider than F9 (**Table 1**). The radicle, scape, pedicel, and flagellomeres F1–F4 of male antennae were significantly wider than those of the corresponding female antennae, but the widths of flagellomeres F5–F9 did not differ significantly between males and females (**Table 1**). The surface area of female antennae (114.17±2.99) ×10^5^ μm^2^ was significantly smaller than that of male antennae (192.50±3.78) ×10^5^ μm^2^ (**Table 1**) (*t*=−16.255, *df*=10, *P*=0.000). There was no significant difference in the area of antennal pedicles between females and males (*t*=0.424, *df*=10, *P*=0.156), whereas the areas of other segments were significantly smaller in females than in males (**Table 1**).

#### 3.1.2 Morphology of maxillary and labial palps

**Figure 2:**
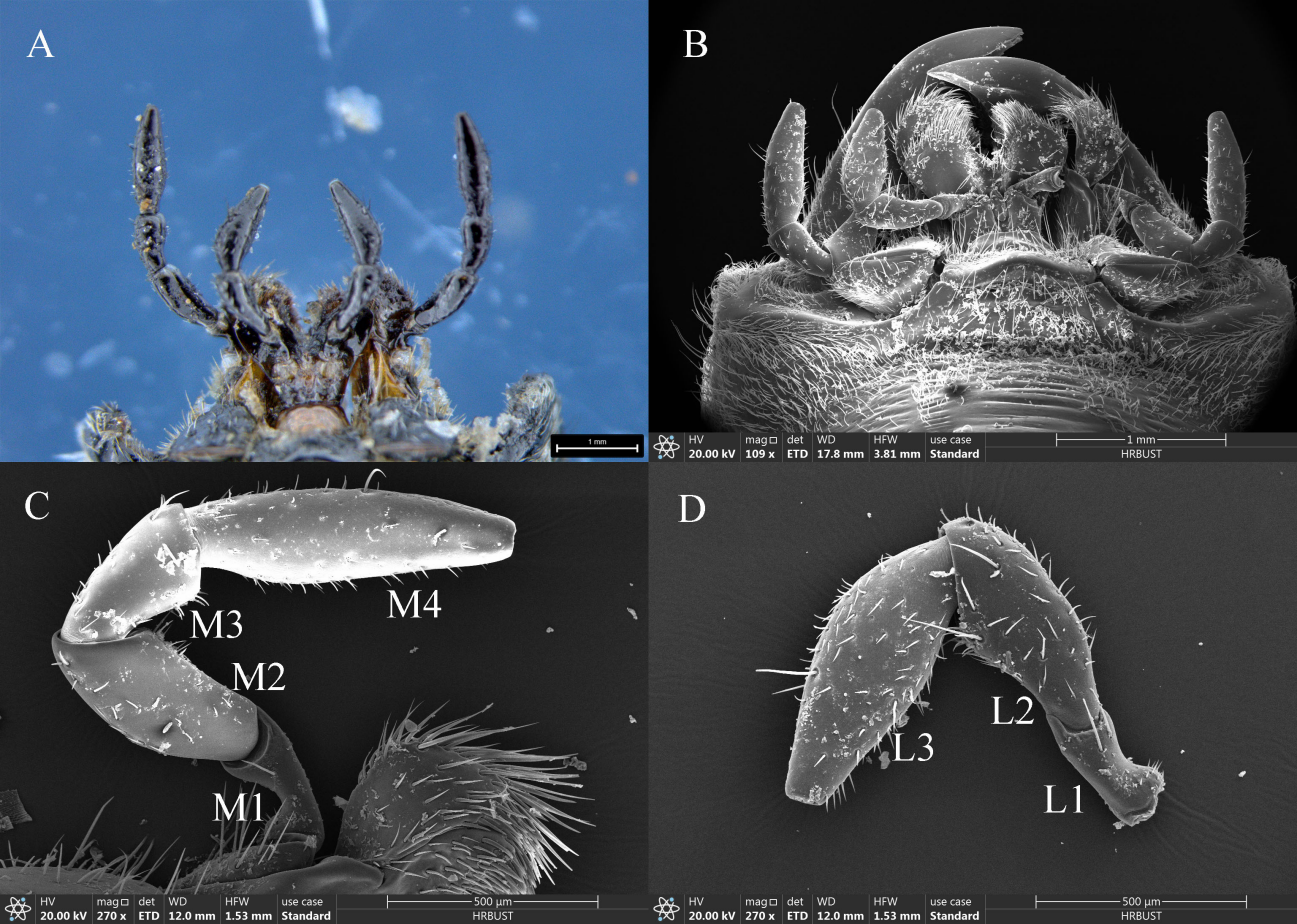
Morphology and structure of the maxillary and labial palps of adult *Mo. saltuarius*. **(A)** Morphology of adult maxillary and labial palps observed under an optical microscope; **(B)** morphology of adult maxillary and labial palps observed by SEM; **(C)** maxillary palp. Abbreviations: M1–M4, first to fourth segments of the maxillary palp; **(D)** labial palp. L1–L3, first to third segments of the labial palp.

The morphology and structural composition of the maxillary and labial palps were the same in males and females. Under the stereomicroscope, both palps were black, with a bright metallic luster (**Figure 2A**); under the electron microscope, sensilla could be seen on their surfaces (**Figure 2B**). The maxillary palps of both males and females comprised 4 segments, M1–M4 (**Figure 2C**). The length of the maxillary palps in females was 1817.77±8.85 μm, and M4 was the longest segment, accounting for 39.26% of the total length. M2, M3, and M1 accounted for 26.13%, 20.97%, and 13.65% of the total length, respectively (**Table 2**). The total length of maxillary palps in males was 1771.98±32.39 μm and did not differ significantly from that in females (*t*=1.363, *df*=10, *P*=0.203). M4 was also the longest segment in males, accounting for 37.80% of the total length, and M2, M3, and M1 accounted for 24.82%, 20.41%, and 16.97%, respectively (**Table 2**). M1 was significantly longer in males than in females (*t*=2.982, *df*=10, *P*=0.014), but none of the other segments differed in length between the sexes (**Table 2**). M2 was the widest maxillary palp segment in females (233.86±4.75 μm) and was 1.72 times wider than the narrowest segment (M1); M2 was also widest in males (223.07±8.73 μm) and was 1.55 times wider than M1. The widths of corresponding maxillary palp segments did not differ significantly between males and females. The M1 surface area was significantly smaller in females than in males (*t*=−5.008, *df*=10, *P*=0.001), but no other maxillary palp segments differed in surface area between the sexes (**Table 2**).

**Table 2:** Characteristics of maxillary palp segments in female and male *Mo. saltuarius*.

Maxillary palp segment	Length (μm)		Width (μm)		Surface area (10^3^ μm^2^)	
	♀	♂	♀	♂	♀	♂
M1	248.08±5.14b	300.62±16.85a	136.20±3.46a	143.66±6.35a	33.74±0.82b	42.78±1.61a
M2	474.94±15.48a	439.76±15.36a	233.86±4.75a	223.07±8.73a	111.30±5.38a	98.53±6.38a
M3	381.14±6.81a	361.74±24.71a	203.27±6.43a	201.34±5.91a	77.44±2.60a	72.87±5.52a
M4	713.61±14.61a	669.87±17.01a	215.78±10.39a	209.34±3.05a	153.45±5.77a	140.02±2.39a
Total	1817.77±8.85a	1771.98±32.39a	—	—	375.93±11.36a	354.20±11.04a

Data are presented as the mean±SE. Within a row, different lowercase letters indicate a significant difference between the sexes (*t*-test, *P*<0.05). *n*=6 per sex. “—” indicates absence.

The labial palps of both males and females comprised 3 segments, L1–L3. The length of female labial palps was 1392.93±31.75 μm; L3 was the longest, accounting for 46.91% of the total length, followed by L2 (36.50%) and L1 (16.59%) (**Table 3**). The length of male labial palps was 1375.34±17.77 μm; L3 was also the longest, accounting for 46.72% of the total length, followed by L2 (35.59%) and L1 (17.69%) (**Table 3**). There were no significant differences in the total length or individual segment lengths of labial palps between females and males (**Table 3**). L3 was the widest segment of female labial palps (240.14±9.32 μm) and was 1.93 times wider than the narrowest segment (L1). L3 was also the widest segment of male labial palps (231.31±4.58 μm) and was 1.65 times wider than L1 (**Table 3**). L1 was significantly narrower in females than in males (*t*=−2.717, *df*=10, *P*=0.022), but the widths of L2 and L3 did not differ between the sexes (**Table 3**). There were no significant differences in the total surface area of labial palps or of individual segments between the sexes (**Table 3**).

**Table 3:** Characteristics of labial palp segments in female and male *Mo. saltuarius*.

Labial palp segment	Length (μm)		Width (μm)		Surface area (10^3^ μm^2^)	
	♀	♂	♀	♂	♀	♂
L1	231.07±9.20a	243.25±10.35a	124.37±5.14b	140.41±2.90a	28.77±1.73a	34.24±2.03a
L2	508.46±20.28a	489.54±16.12a	215.62±2.85a	216.38±5.69a	109.62±4.57a	105.79±3.67a
L3	653.41±10.54a	642.55±13.91a	240.14±9.32a	231.31±4.58a	157.03±7.12a	148.86±5.67a
Total	1392.93±31.75a	1375.34±17.77a	—	—	295.42±9.41a	288.89±7.47a

Data are presented as the mean±SE. Within a row, different lowercase letters indicate a significant difference between the sexes (*t*-test, *P*<0.05). *n*=6 per sex. “—” indicates absence.

### 3.2 Types and distribution of antennal sensilla

Both sexes had 8 types of sensilla on their antennae: Böhm bristles (BBs), sensilla trichodea (STs, with subtypes STI, STII and STIII), sensilla chaetica (SChs, including SChI and SChII subtypes), sensilla auricillica (SAus), sensilla basiconica (SBs, including SBI and SBII subtypes), sensilla grooved peg (SGPs), dome shaped organs (DSOs), and cuticular pores (CPs). The shapes and distributions of various sensilla are described below.

#### 3.2.1 Böhm bristles

**Figure 3:**
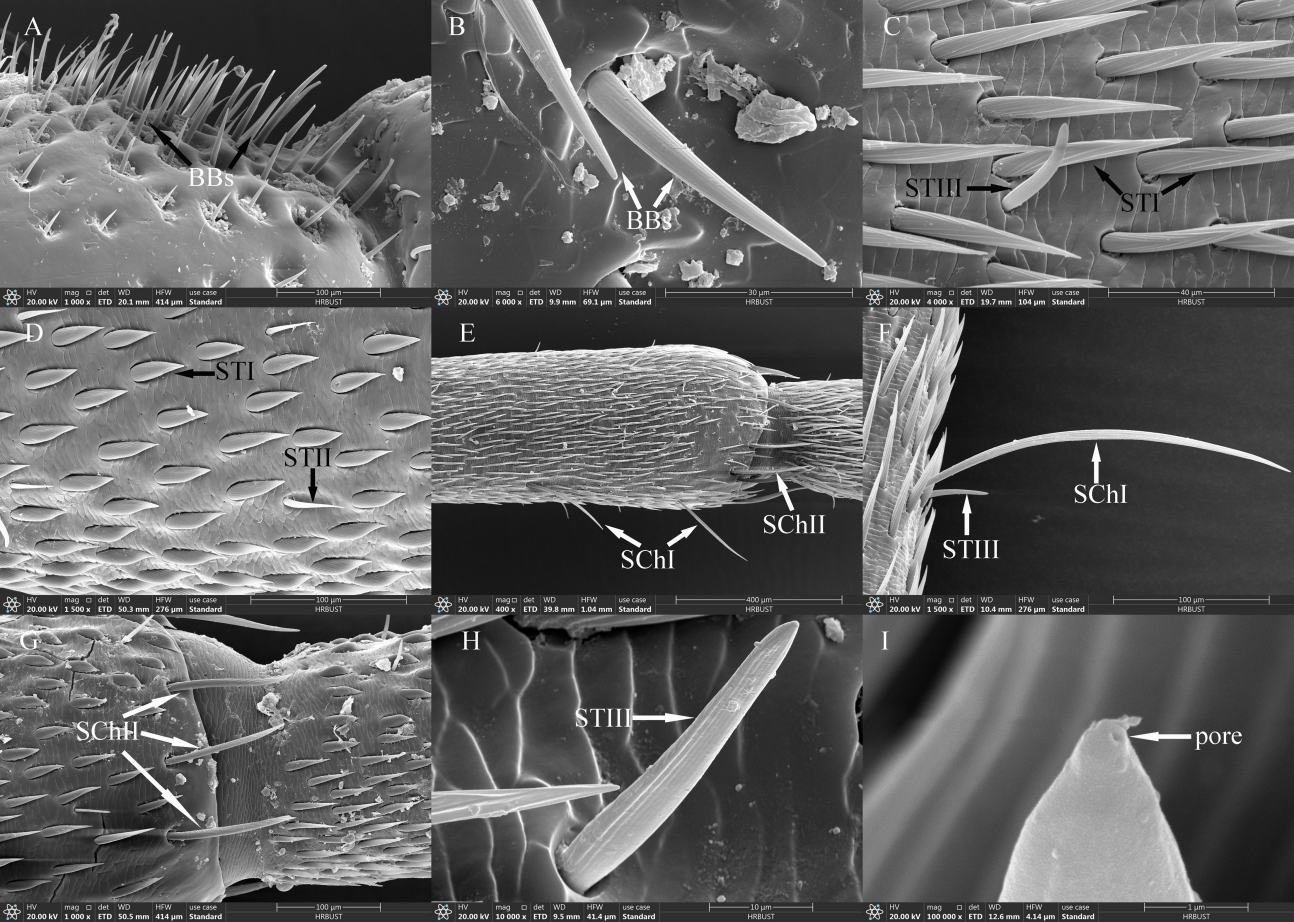
Ultrastructure of antennal sensilla in adult *Mo. saltuarius*. **(A)** BBs on the antennifer; **(B)** BBs with longitudinal lines on the side wall; **(C)** STIs and STIIIs on male antennae; **(D)** STIs and STIIs on male antennae; **(E)** SChIs and SChIIs; **(F)** SChIs and STIIIs; **(G)** SChIIs; **(H)** STIIIs with longitudinal lines on the side wall; **(I)** terminal pore of STIII.

BBs were conical and straight, standing almost perpendicular to the surface of the antennae. They had shallowly grooved hairs and sharp tips; emerged from flexible, shallow cuticular sockets; and lacked visible pores (**Figures 3A, B**). BBs on the female antennae were 58.82±4.13 μm long (*n=*100) with a basal diameter of 5.82±0.18 μm (*n=*100); those on male antennae were 61.09±3.30 μm long (*n=*100) with a basal diameter of 6.60±0.14 μm (*n=*100) (**Table 4**). Clusters of BBs were visible on the radicle and pedicel bases of both sexes (**Table 5**), and there were no significant differences in BB density between males and females (**Table 5**).

**Table 4:** Size and morphological characteristics of antennal sensilla in *Mo. saltuarius*.

Sensillum type/subtype	Length (μm)		Basal diameter (μm)		Tip	Wall	Shape	Socket	Pores
	♀	♂	♀	♂					
BB	58.82±4.13a	61.09±3.30a	5.82±0.18b	6.60±0.14a	Sharp	Grooved	Straight	Flexible	No pore
STI	48.55±1.57a	43.62±0.61b	5.77±0.09b	7.93±0.26a	Sharp	Grooved	Straight or curved	Flexible	No pore
STII	49.03±1.50a	43.97±1.86b	4.15±0.07a	4.23±0.09a	Sharp	Grooved	Straight or slightly curved	Flexible	No pore
STIII	38.72±1.66a	30.58±1.11b	4.27±0.08a	3.90±0.08b	Dullish	Grooved	Straight or inversely curved	Flexible	Terminal pore
SChI	189.35±3.92a	172.32±5.46b	7.37±0.22a	6.06±0.17b	Sharp	Grooved	Straight or curved	Flexible	No pore
SChII	152.03±4.12a	149.48±4.81a	8.64±0.19b	10.27±0.18a	Sharp	Grooved	Straight	Flexible	No pore
SAu	11.34±0.14b	11.96±0.15a	3.24±0.04a	3.09±0.03b	Dullish	Cuticular pores	Straight	Inflexible	Multiporous
SBI	17.29±0.23a	16.55±0.26b	2.92±0.04a	2.38±0.03b	Dullish	Cuticular pores	Straight or slightly curved	Inflexible	Multiporous?
SBII	7.22±0.27a	5.74±0.13b	3.06±0.20a	2.96±0.08a	Dullish	Cuticular pores	Straight	Inflexible	Terminal pore
SGP	6.21±0.36a	6.10±0.32a	2.45±0.10a	2.93±0.20a	Dullish	Smooth +Grooved	Straight	Inflexible	Multiporous?
DSO	9.83±0.20a	9.45±0.15a	2.87±0.04a	2.83±0.05a	Blunt	Smooth	Straight	—	Terminal pore
CP	—	—	1.29±0.22a	0.87±0.07a	—	—	—	—	—

Data are presented as mean±SE. Within each row, different lowercase letters indicatesignificant differences between the sexes (*t*-test, *P*<0.05).?: indicates that this sensillum is hypothesized to have pores, but they were not clearly visible in the studied material.

**Table 5:** Densities and distribution of antennal sensillum types/subtypes in male and female *Mo. saltuarius*.

Segment		Sex	BB	STI	STII	STIII	SChI	SChII	SAu	SBI	SBII	SGP	DSO	CP	Total
Antennifer		♀	66.12±6.02a	—	—	—	—	—	—	—	—	—	—	11.45±2.43a	77.57±6.21a
		♂	51.95±3.42a	—	—	—	—	—	—	—	—	—	—	6.03±2.79a	57.98±4.98b
Scape		♀	—	62.26±4.65a	—	1.97±0.83a	—	—	—	—	—	—	—	9.67±2.89a	73.91±7.00a
		♂	—	36.54±4.44b	—	3.22±0.49a	—	—	—	—	—	—	—	10.07±2.74a	49.83±4.14b
Pedicel		♀	13.45±4.15a	86.14±6.25a	—	2.67±0.41a	—	—	—	—	—	—	—	3.75±2.49a	106.01±8.57a
		♂	5.68±1.80a	80.13±2.46a	—	2.60±0.36a	—	—	—	—	—	—	—	0.82±0.20a	89.23±1.23a
Flagellum	F1	♀	—	133.86±11.10a	0.56±0.40b	2.73±0.59a	0.21±0.06b	0.40±0.04a	0.46±0.23a	0.16±0.16a	—	—	0.05±0.05	6.13±3.07a	144.57±8.09a
		♂	—	52.65±2.47b	7.17±1.16a	3.16±0.42a	0.63±0.03a	0.15±0.02b	2.30±1.45a	0.07±0.07a	0.07±0.07	—	—	11.85±4.23a	77.48±2.69b
	F2	♀	—	163.40±7.10a	0.45±0.38b	3.57±0.44a	0.26±0.06a	0.67±0.05a	11.91±11.78a	5.40±5.40a	—	—	1.55±1.55	5.55±3.49a	192.76±20.26a
		♂	—	62.93±8.67b	5.24±0.82a	2.79±0.34a	0.05±0.03b	0.21±0.04b	8.45±5.11a	10.32±5.16a	0.11±0.11	—	—	19.68±5.36a	109.78±13.88b
	F3	♀	—	174.78±8.03a	0.54±0.21b	4.14±0.38a	0.17±0.04a	0.69±0.04a	10.71±9.37a	8.26±6.09a	—	—	0.39±0.39a	9.02±2.64b	208.69±18.48a
		♂	—	76.28±9.38b	4.73±0.37a	3.96±0.59a	0.05±0.02b	0.26±0.04b	9.50±5.60a	15.47±7.40a	0.29±0.22	0.11±0.11	0.05±0.05a	26.16±3.81a	136.84±19.25b
	F4	♀	—	181.52±7.79a	0.99±0.49b	3.65±0.40a	0.19±0.05a	0.76±0.05a	8.93±7.32a	5.33±3.88a	—	0.39±0.39a	0.55±0.55a	8.55±1.87b	210.85±17.40a
		♂	—	89.87±16.43b	5.58±0.61a	3.82±0.56a	0.10±0.03a	0.27±0.06b	17.09±7.58a	10.62±4.82a	0.20±0.13	0.20±0.20a	0.05±0.05a	28.41±4.83a	156.22±25.82a
	F5	♀	—	185.35±15.32a	1.83±1.07b	4.43±0.63a	0.21±0.02a	0.84±0.06a	6.81±1.76a	5.29±1.98a	0.05±0.05a	—	0.07±0.07a	10.31±2.19b	215.20±18.44a
		♂	—	97.70±12.28b	4.78±0.56a	4.10±0.53a	0.09±0.02b	0.42±0.01b	13.94±6.05a	11.41±5.19a	0.24±0.13a	0.54±0.29	0.07±0.07a	29.62±4.78a	162.90±18.40a
	F6	♀	—	215.75±10.43a	1.80±0.90a	5.43±0.78a	0.16±0.08a	1.12±0.10a	17.86±5.26a	13.04±5.37a	0.06±0.06a	—	—	15.35±3.63a	270.60±18.25a
		♂	—	100.47±22.31b	4.67±1.09a	3.95±0.73a	0.13±0.03a	0.52±0.07b	25.57±10.83a	21.47±10.82a	0.55±0.29a	0.64±0.52	0.12±0.08	39.08±10.56a	197.16±38.30a
	F7	♀	—	254.26±31.93a	2.17±1.15a	7.74±1.50a	0.26±0.07a	1.13±0.11a	31.05±8.30a	23.83±15.36a	0.20±0.20a	0.13±0.13a	0.26±0.20a	12.07±1.61b	333.10±55.17a
		♂	—	112.16±19.67b	3.91±0.83a	4.69±0.76a	0.17±0.03a	0.64±0.08b	28.00±8.72a	21.05±7.82a	0.26±0.20a	0.07±0.07a	0.16±0.16a	39.74±7.33a	210.85±29.55a
	F8	♀	—	303.84±34.83a	2.85±1.42a	7.41±1.24a	0.15±0.05a	1.40±0.08a	52.84±8.15a	34.07±10.17a	0.25±0.13a	0.57±0.32a	1.39±0.38a	14.05±5.07a	418.82±49.51a
		♂	—	149.45±15.81b	5.12±0.75a	5.52±0.63a	0.17±0.04a	0.71±0.02b	40.46±7.91a	26.63±6.48a	0.62±0.15a	0.50±0.19a	0.31±0.21b	31.73±6.79a	261.22±22.28b
	F9	♀	—	328.04±6.07a	2.61±1.65a	14.75±3.20a	0.14±0.07a	—	57.28±12.54a	35.33±3.82a	0.60±0.13a	0.64±0.31a	0.58±0.19a	6.43±2.12a	446.40±14.96a
		♂	—	257.48±14.52b	5.38±1.00a	22.33±3.70a	0.09±0.03a	—	35.89±5.55a	20.61±4.51b	1.23±0.31a	0.84±0.32a	0.52±0.17a	19.34±5.76a	363.70±21.20b

Data are presented as the mean±SE. Within a column, different lowercase letters indicate significant differences between the sexes (*t*-test, *P*<0.05). Density is recorded as the number per 10^5^ μm^2^. *n*=6 per sex. “—” indicates absence.

#### 3.2.2 Sensilla trichodea

STs were divided into three subtypes, STIs, STIIs and STIIIs. These sensilla lay flat against the surface of the antenna with their tips oriented towards the apex. STIs on female antennae were stout and bristle-like with broad sockets, pointed tips, and grooved walls; they could be either straight or curved, with no wall or apical pores (**Figure 3C**). STIs on male antennae resembled bamboo shoots, with a thick base that extended uniformly towards the distal end and contracted sharply at the tip, ending in a narrow point (**Figure 3D**). STIs on female antennae were 48.55±1.57 μm long (*n=*60) and were significantly longer (*t*=3.413, *df*=158, *P*=0.001) than those on male antennae (43.62±0.61 μm, *n=*100). The basal diameter of STIs on female antennae was 5.77±0.09 μm (*n=*60), significantly smaller (*t*=-6.279, *df*=158, *P*=0.000) than that on male antennae (7.93±0.26 μm) (*n=*100; **Table 4**). Each STII appeared overall trichoid in shape, with its base attached to the basal socket and standing straight or slightly curved; it exhibited longitudinal or oblique grooves on its surface and terminated in a sharp point at the tip (**Figure 3D**). STIIs on female antennae were 49.03±1.50 μm long (*n=*60), significantly longer (*t*=2.140, *df*=108, *P*=0.035) than those on male antennae (43.97±1.86 μm) (*n=*50; **Table 4**). The basal diameter of STIIs on female antennae was 4.15±0.07 μm (*n=*60) and did not differ significantly from that on male antennae (4.23±0.09 μm) (*n=*50; **Table 4**). STIIIs were located in slightly raised basal sockets on the antennal surface, oriented perpendicular to the surface and either standing upright or curved inward. Their surfaces exhibited longitudinal grooves, and their slightly blunt apices contained a terminal pore (**Figures 3C, H, I**). In female antennae, STIIIs measured 38.72±1.66 μm in length (*n=*60) with a basal diameter of 4.27±0.08 μm (*n=*60); both values were significantly greater than those of males (*t_1_ =* 3.917, *df_1_ =* 108, *P_1_ =* 0.000; *t_2_ =* 3.279, *df_2_ =* 108, *P_2_ =* 0.001), whole STIIIs measured 30.58±1.11 μm in length (*n=*50) and 3.90±0.08 μm in basal diameter (*n=*50; **Table 4**).

STIs were widely distributed and most densely populated on the antennae of both sexes, where they were present on the scape, pedicel, and all flagellomeres. On female antennae, STI density increased from the base to the tip, with the highest density on the terminal flagellomere. On male antennae, STI density initially increased from the base to the tip, then decreased before increasing again, with a maximum density on F9. STI densities on the scape were 62.26±4.65 per 10^5^ μm^2^ in females and 36.54±4.44 per 10^5^ μm^2^ in males, and STI densities on F9 were 328.04±6.07 per 10^5^ μm^2^ in females and 257.48±14.52 per 10^5^ μm^2^ in males. STI density on the pedicel did not differ significantly between female and male antennae, but STI densities on the scape and all flagellomeres were significantly greater in females than in males (**Table 5**). STIIs were found across all flagellomeres in both female and male antennae, and their densities on F1–F5 were significantly lower in females than in males; however, STIIs on flagellomeres F6–F9 did not differ significantly between the sexes (**Table 5**). STIIIs were present across all antennal segments—scape, pedicel, and flagellum—in both sexes; their densities increased from the base toward the tip and showed no significant differences between corresponding segments of males and females. In female antennae, STIIIs were less abundant on the scape (1.97±0.83 per 10^5^ μm^2^) and most abundant on F9 (14.75±3.20 per 10^5^ μm^2^). In males, the lowest density occurred on the pedicel (2.60±0.36 per 10^5^ μm^2^) and the highest on F9 (22.33±3.70 per 10^5^ μm^2^; **Table 5**).

#### 3.2.3 Sensilla chaetica

SChs were classified into two subtypes, SChIs and SChIIs. SChIs were slender and typically curved inward. They arose form sockets and projected almost perpendicularly from the antennal surface, with deeply grooved longitudinal walls and sharply pointed tips (**Figures 3E, F**). SChIs on female antennae were 189.35±3.92 μm long (*n=*60) with a basal diameter of 7.37±0.22 μm (*n=*60); they were significantly longer than SChIs on male antennae (172.32±5.46 μm) (*n=*50) and had greater basal diameters than those of males (6.06±0.17 μm) (*n=*50) (*t_1_ =* 4.600, *df_1_ =* 108, *P_1_ =* 0.000; *t_2_ =* 2.589, *df_2_ =* 108, *P_2_ =* 0.011; **Table 4**). SChIIs had a straight shape and were thicker than SChIs. Each was parallel to the antennal surface, with its base located in a deeper socket; it had deep longitudinal grooves on its surface, along with a sharp tip (**Figures 3E, G**). SChIIs on female antennae were 152.03±4.12 μm long (*n=*60) and were not significantly longer (*t*=0.406, *df*=108, *P*=0.686) than those on male antennae (149.48±4.81 μm). The basal diameter of SChIIs on female antennae was 8.64±0.19 μm (*n=*60) and was significantly smaller (*t*=−6.093, *df*=108, *P*=0.000) than that on male antennae (10.27±0.18 μm, *n=*50; **Table 4**).

SChIs were found exclusively on the flagellum of both female and male antennae and were present on flagellomeres F1–F9, albeit in relatively low numbers. Notably, the density of SChIs on F1 was significantly lower in females than in males. Conversely, the densities of SChIs on F2, F3, and F5 were significantly higher in females than in males. There were no significant differences in SChI density between the sexes on other flagellomeres (**Table 5**). SChIIs were found on F1–F8 of both female and male flagella. SChII density increased progressively from the base to the tip in antennae of both sexes. SChII density on each flagellomere was significantly higher in females than in males. The lowest SChII density recorded in females was 0.40±0.04 per 10^5^ μm^2^ on F1, and the highest was 1.40±0.08 per 10^5^ μm^2^ on F8; by contrast, the lowest SChII density in males was 0.15±0.02 per 10^5^ μm^2^ (F1), and the highest was 0.71±0.02 per 10^5^ μm^2^ (F8) (**Table 5**).

#### 3.2.4 Sensilla auricillica

**Figure 4:**
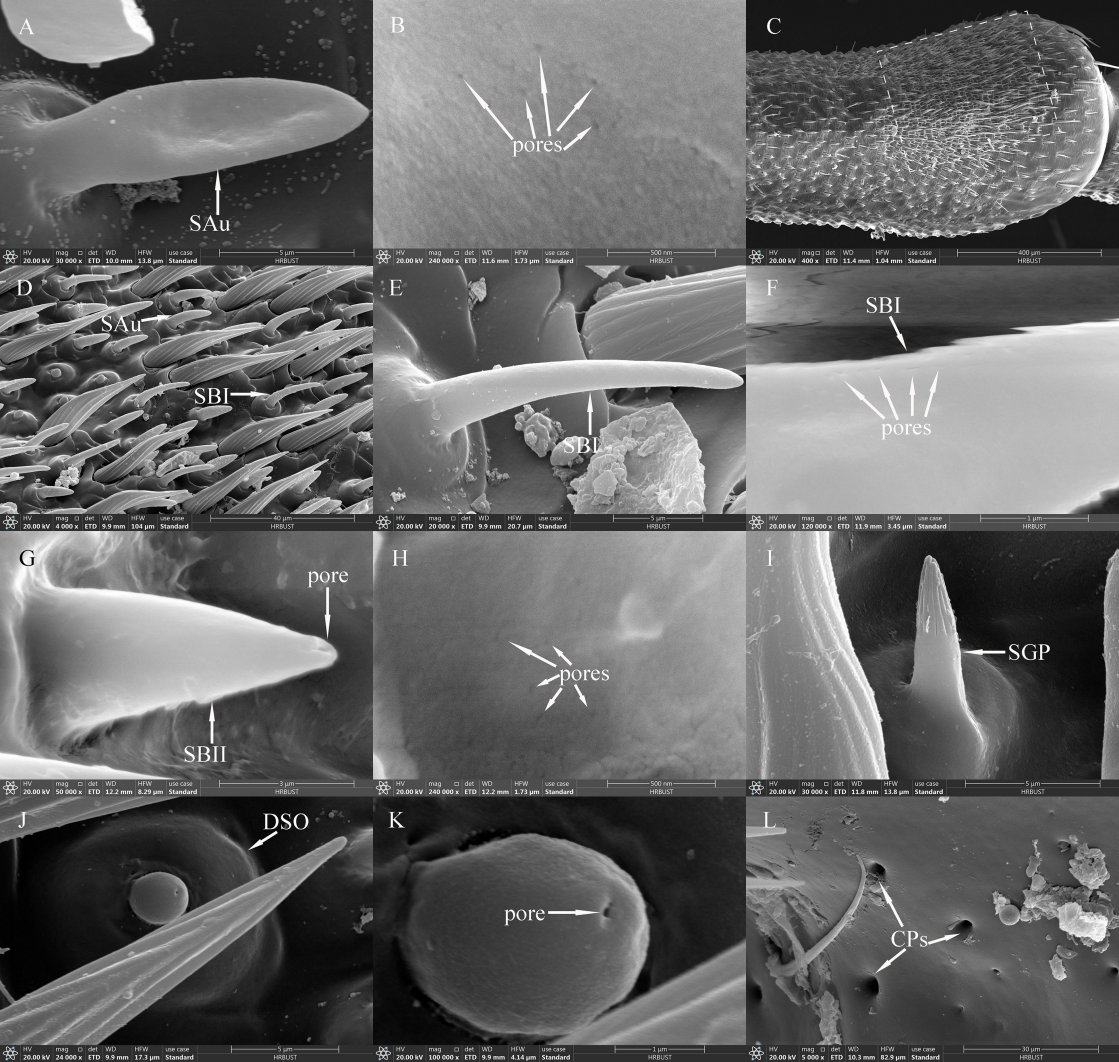
SAus, SBIs, SBIIs, SGPs, DSOs and CPs of adult *Mo. saltuarius*. **(A)** SAu; **(B)** cuticular pores of SAu; **(C)** the distal end of male antenna F6, with the dashed-line area indicating dense sensilla distribution; **(D)** area of dense sensilla, with highlighted SAu and SBI; **(E)** SBI; **(F)** cuticular pores of SBI; **(G)** SBII and its terminal pores; **(H)** cuticular pores of SBII; **(I)** SGP; **(J)** DSO; **(K)** terminal pore of DSO; **(L)** CPs.

SAus resembled a rabbit’s ear in shape, with a multiporous surface and relatively dull tips (**Figures 4A, B**). They were cylindrical at the base, with inflexible, shallow sockets, and were almost perpendicular to the antennal surface. SAus on female antennae were 11.34±0.14 μm long (*n*=60), significantly smaller (*t*=−3.027, *df*=108, *P*=0.003) than those on male antennae (11.96±0.15 μm) (*n*=50). The basal diameter of SAus on female antennae was 3.24±0.04 μm (*n*=60), significantly larger (*t*=3.096, *df* =108, *P*=0.002) than that on male antennae (3.09±0.03 μm) (*n*=50). SAu were found across all flagellomeres in both males and females, and their densities on individual flagellomeres did not differ between the sexes. SAus are mainly found in areas of dense sensilla distribution on each flagellomere (**Figures 4C, D**). On female antennae, SAu density initially increased from the base toward the tip, then decreased, and finally increased again. SAu density was lowest on F1 (0.46±0.23 per 10^5^ μm^2^) and highest on F9 (57.28±12.54 per 10^5^ μm^2^). On male antennae, SAu density initially increased from the base toward the tip, then decreased, increased, and decreased again. SAu density was lowest on F1 (2.30±1.45 per 10^5^ μm^2^) and highest on F8 (40.46±7.91 per 10^5^ μm^2^) (**Table 5**).

#### 3.2.5 Sensilla basiconica

SBs on *Mo. saltuarius* antennae were divided into two subtypes, SBIs and SBIIs. SBIs were slender and conical; they lacked a basal socket and curved towards a relatively dull antennal tip; the presence of pores was hypothesized, but they were not clearly visible in the studied material (**Figures 4E, F**). SBIs on female antennae were 17.29±0.23 μm long (*n=*60) with a basal diameter of 2.92±0.04 μm (*n=*60); these values were both significantly larger than those of SBIs on male antennae (16.55±0.26 μm in length and 2.38±0.03 μm in basal diameter, *n=*50) (**Table 4**). SBIIs were short and conical with a relatively dull tip that featured a terminal pore; the presence of pores was hypothesized, but they were not clearly visible in the studied material; they stood upright and perpendicular to the antennal surface (**Figures 4G, H**). SBIIs on female antennae were 6.87±0.39 μm long (*n=*15), significantly longer than those on male antennae (5.62±0.14 μm) (*n=*38). However, there was no significant difference in SBII basal diameter between female antennae (3.06±0.15 µm, *n=*15) and male antennae (2.97±0.09 µm, *n=*38). SBIs were found on all flagellomeres, and their densities on flagellomeres F1–F8 did not differ among the sexes. However, the SBI density on F9 was significantly greater in females than in males. On female antennae, SBI density initially increased from the base toward the tip, then decreased, and finally increased again; it was lowest on F1 (0.16±0.16 per 10^5^ μm^2^) and highest on F9 (35.33±3.82 per 10^5^ μm^2^). On male antennae, SBI density initially increased from the base toward the tip, then decreased, increased, and decreased again; it was lowest on F1 (0.07±0.07 per 10^5^ μm^2^) and highest on F8 (26.63±6.48 per 10^5^ μm^2^, **Table 5**). SBII were found on F5–F9 of female antennae and F1–F9 of male antennae. Their density on F5–F9 did not differ between the sexes. SBII density increased from F5 (0.05±0.05 per 10^5^ μm^2^) to F9 (0.60±0.13 per 10^5^ μm^2^) on female antennae and from F1 (0.07±0.07 per 10^5^ μm^2^) to F9 (1.23±0.31 per 10^5^ μm^2^; **Table 5**) on male antennae.

#### 3.2.6 Sensilla grooved peg

SGPs were distinctive double-walled sensilla, smooth basally and grooved distally, that stood upright and perpendicular to the antennal surface; they emerged from a raised cuticular area and had a non-articulating base (**Figure 4I**). SGPs on female antennae were 6.21±0.36 μm long (*n=*11) with a basal diameter of 2.45±0.10 μm (*n=*11), and those on male antennae were 6.10±0.32 μm long (*n=*12) with a basal diameter of 2.93±0.20 μm (*n=*12, **Table 4**); neither their length nor their diameter differed between the sexes (*t_1_ =* 0.234, *df_1_ =* 21, *P*=0.817; *t_2_* =−2.118, *df_2_ =* 21, *P*=0.052). SGPs were found on F4 and F7–F9 of female antennae, and their density first decreased and then increased across these four flagellomeres. SGP density was lowest on F7 (0.13±0.13 per 10^5^ μm^2^) and highest on F9 (0.64±0.31 per 10^5^ μm^2^). SGPs were found on F3–F9 of male antennae. Their density initially increased towards the tip, then decreased, and finally increased again; it was lowest on F7 (0.07±0.07 per 10^5^ μm^2^), and highest on F9 (0.84±0.32 per 10^5^ μm^2^; **Table 5**).

#### 3.2.7 Dome shaped organs

DSOs were small, round sensilla that emerged from elevated cuticular domes on the antennal surface. Each DSO had a central terminal pore, sometimes surrounded by a raised cuticular collar (**Figures 4J, K**). On female antennae, the diameter of the DSO base was 9.83±0.20 μm (*n=*28), and the diameter of the semi-spherical structure was 2.87±0.04 μm (*n=*28). These measurements did not differ significantly from those of males, which were 9.45±0.15 μm (*n*=22) and 2.83±0.05 μm (*n*=22), respectively (*t_1_ =* 1.443, *df_1_ =* 48, *P_1_ =* 0.155; *t_2_ =* 0.640, *df_2_ =* 48, *P_2_ =* 0.525; **Table 4**). DSOs were found on the antennal flagellum in both males and females. In females, DSOs were present on F1–F5 and F7–F9 but not on F6. Their density increased, decreased, and increased again from the base to the tip and was highest on F8 (1.55±1.55 per 10^5^ μm^2^). In males, DSOs were found on F3–F9; their density was similar on F3 and F4 (0.05±0.05 per 10^5^ μm^2^), then gradually increased from F5 to F9 (0.52±0.17 per 10^5^ μm^2^). DSO density on F8 was higher in females than in males, but no other flagellomeres showed differences in DSO density between the sexes (**Table 5**).

#### 3.2.8 Cuticular pores

CPs were visible as small openings in the epidermal surface (**Figure 4L**); their diameters were 1.29±0.22 μm in females (*n=*20) and 0.87±0.07 μm in males (*n=*19), and these values did not differ significantly (*t*=1.756, *df*=37, *P*=0.087; **Table 4**).CPs were present on the antennifer, scape, pedicel, and all flagellomeres in both females and males. Their densities on the antennifer, scape, pedicel, F1, F2, F6, F8, and F9 did not differ significantly between the sexes, whereas densities on F3, F4, F5, and F7 were significantly lower in females (**Table 5**).

#### 3.2.9 Distribution of sensilla on the antennae

The total densities of antennal sex in both males and females increased progressively from the base to the tip, with the highest density observed on the most distal flagellomere (**Table 5**). Both female and male *Mo. saltuarius* had two types of sensillum on their antennifers: BBs and CPs, which accounted for 85.24% and 14.76% of the sensilla on female antennifers and 89.60% and 10.40% of the sensilla on male antennifers, respectively. Both females and males had three types/subtypes of sensillum on their scapes: STIs, CPs, and STIIIs, which accounted for 84.24%, 13.09%, and 2.67% of the sensilla on female scapes and 73.32%, 20.21%, and 6.47% of the sensilla on male scapes. Both females and males had four types/subtypes of sensillum on their pedicels: STIs, BBs, CPs, and STIIIs, which accounted for 81.26%, 12.68%, 3.54%, and 2.51% of the sensilla on female pedicels and 89.80%, 6.36%, 0.92%, and 2.91% of the sensilla on male pedicels.

In female antennae, there were nine sensillum types/subtypes on F1, F2, and F3: CPs, STIIIs, STIs, STIIs, ChIs, ChIIs, SAus, SBIs, and DSOs. The five most common types/subtypes on F1 were STIs (92.59%), CPs (4.24%), STIIIs (1.89%), STIIs (0.39%), and SAus (0.32%); on F2, they were STIs (84.77%), SAus (6.18%), CPs (2.88%), SBIs (2.80%), and STIIIs (1.85%); and on F3, they were STIs (83.75%), SAus (5.13%), CPs (4.32%), SBIs (3.96%), and STIIIs (1.98%). F4 had one more type of sensillum than F3, namely SGPs; its five most common types/subtypes were STIs (86.09%), SAus (4.23%), CPs (4.05%), SBIs (2.53%), and STIIIs (1.73%). F5 had one more subtype of sensillum than F4, namely SBII, but it lacked SGP; its five most common types/subtypes were STIs (86.13%), CPs (4.79%), SAus (3.16%), SBIs (2.46%), and STIIIs (2.06%). Unlike F5, F6 lacked DSOs; its five most common types/subtypes were STIs (79.73%), SAus (6.60%), CPs (5.67%), SBIs (4.82%), and STIIIs (2.01%). F7 and F8 had two more types of sensillum than F6, namely DSOs and SGPs. The five most common types/subtypes on F7 were STIs (76.33%), SAus (9.32%), SBIs (7.16%), CPs (3.62%), and STIIIs (2.32%); on F8, they were STIs (72.55%), SAus (12.62%), SBIs (8.13%), CPs (3.35%), and STIIIs (1.77%). Unlike F8, F9 lacked SChII; its five most common types/subtypes were STIs (73.48%), SAus (12.83%), SBIs (7.91%), STIIIs (3.30%), and CPs (1.44%).

In male antennae, there were nine types/subtypes of sensillum on F1 and F2: CPs, STIIIs, STIs, STIIs, ChIs, ChIIs, SAus, SBIs, and SBIIs. The five most common types/subtypes on F1 were STIs (67.95%), CPs (15.29%), STIIs (9.26%), STIIIs (4.08%), and SAus (2.97%); on F2, they were STIs (57.32%), CPs (17.93%), SBIs (9.40%), SAus (7.69%), and STIIs (4.78%). There were two additional types of sensillum on F3–F8: DSO and SGP. The five most common types/subtypes on F3 were STIs (55.74%), CPs (19.12%), SBIs (11.30%), SAus (6.94%), and STIIs (3.45%); on F4, they were STIs (57.53%), CPs (18.18%), SAus (10.94%), SBIs (6.80%), and STIIs (3.57%); on F5 they were STIs (59.98%), CPs (18.19%), SAus (8.55%), SBIs (7.00%), and STIIs (2.93%); on F6 they were STIs (50.96%), CPs (19.82%), SAus (12.97%), SBIs (10.89%), and STIIs (2.37%); on F7, they were STIs (53.19%), CPs (18.85%), SAus (13.28%), SBIs (9.98%), and STIIIs (2.23%); and on F8, they were STIs (57.21%), SAus (15.49%), CPs (12.15%), SBIs (10.19%), and STIIIs (2.11%). F9 had one fewer subtype of sensillum than F8, and its five most common types/subtypes were STIs (70.79%), SAus (9.87%), STIIIs (6.14%), SBIs (5.67%), and CPs (5.32%).

The numbers of several antennal sensilla types/subtypes showed significant sexual dimorphism in adult *Mo. saltuarius* (**Table 6**). Four types/subtypes—BB, STI, SChII, and DSO—were significantly more numerous in females, whereas eight others—STII, STIII, SChI, SAu, SBI, SBII, SGP, and CP—were significantly less numerous in females than in males (**Table 6**).

**Table 6:** Number of antennal sensillum types/subtypes in male and female *Mo. saltuarius.*

Sensillum type/subtype	Total number	
	♀	♂
BB	299.81± 11.76a	242.43± 7.04b
STI	17842.04±512.53a	15006.56±262.99b
STII	96.99±2.78b	877.28±12.96a
STIII	459.26±13.48b	816.24±17.70a
SChI	17.88±0.54b	37.18±0.80a
SChII	60.19±1.70a	45.18±0.51b
SAu	1232.24±38.75b	2143.16±34.93a
SBI	797.53±24.41b	1751.52±22.63a
SBII	5.76±0.20b	41.55±0.85a
SGP	9.90±0.34b	30.74±0.83a
DSO	45.37±1.65a	12.14±0.30b
CP	981.99±23.94b	3974.44±57.18a
Total	21848.95±621.58b	24978.44±403.45a

Data are presented as mean±SE. The number of each sensillum type/subtype was calculated by multiplying the area of each segment by the corresponding sensillum density and summing the results. Within each row, different lowercase letters indicate significant differences between the sexes (*t*-test, *P*<0.05). *n*=6 per sex.

### 3.3 Types and distribution of sensilla on the labial and maxillary palps

Both male and female *Mo. saltuarius* had 7 types of sensilla on their labial and maxillary palps: BBs, STs (including STII and STIII subtypes), SChs, SPs, SCos, CPs, and STBs (including the STBI, STBII, STBIII, and STBIV subtypes). The morphologies, numbers, and distribution of these sensilla are described below.

#### 3.3.1 BBs

**Figure 5:**
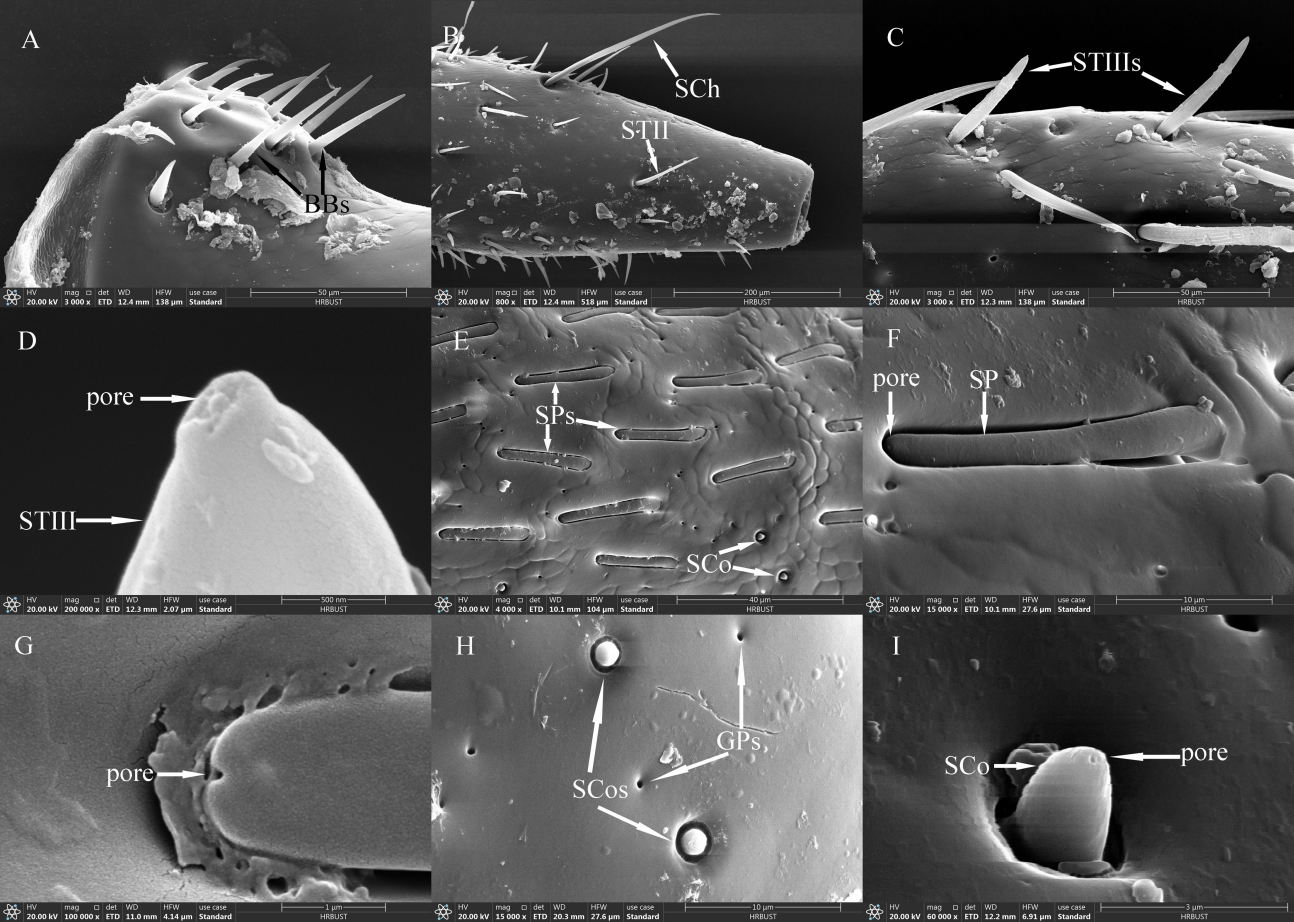
Ultrastructure of sensilla on maxillary and labial palps of adult *Mo. saltuarius*. **(A)** BBs on a maxillary palp; **(B)** STII and SChs; **(C)** STIIIs; **(D)** terminal pore of an STIII; **(E)** morphological characteristics of SPs; **(F, G)** terminal pores of SPs; **(H)** SCos and CPs; **(I)** terminal pore of an SCo.

BBs were present on the maxillary and labial palps of adult *Mo. saltuarius* and were morphologically similar to those on the adult antennae (**Figure 5A**). These conical, straight sensilla had sharp tips and smooth-walled hairs; they emerged from shallow flexible cuticular sockets and were nearly perpendicular to the palp surface. BBs on female maxillary palps were 18.47±1.18 μm long (*n=*13) with a basal diameter of 3.45±0.16 μm (*n=*13); these values did not differ significantly from those of BBs on male maxillary palps (*t_1_ =* 1.736, *df_1_ =* 21, *P_1_ =* 0.097; *t_2_ =* 0.898, *df_2_ =* 21, *P_2_ =* 0.379; **Table 7**), which were 15.74±0.92 μm long (*n=*10) with a basal diameter of 3.25±0.18 μm (*n=*10). BBs on female labial palps were 19.42±1.22 μm long (*n=*20) with a basal diameter of 3.83±0.13 μm (*n=*20); again, these values did not differ significantly from those of BBs on male labial palps (*t_1_* =−1.463, *df_1_ =* 40, *P_1_ =* 0.151; *t_2_* =−0.525, *df_2_ =* 40, *P_2_ =* 0.603), which were 22.18±1.41 μm long (*n=*22) with a basal diameter of 3.91±0.11 μm (*n=*22). BBs were found only on M1 and L1 in both males and females, and the number of BBs did not differ between the sexes (**Table 8**).

**Table 7:** Size and morphological characteristics of sensilla on the maxillary and labial palps of adult *Mo. saltuarius.*.

Sensillum type/ subtype	Organ	Length (μm)		Basal diameter (μm)		Tip	Wall	Shape	Socket	Pores
		♀	♂	♀	♂					
BB	MP	18.47±1.18a	15.74±0.92 a	3.45±0.16a	3.25±0.18a	Sharp	Grooved	Straight	Flexible	No pore
	LP	19.42±1.22a	22.49±1.87a	3.83±0.13a	3.76±0.12a					
STII	MP	38.75±0.98a	41.85±1.84a	3.99±0.08b	4.29±0.09a	Sharp	Grooved	Straight	Flexible	No pore
	LP	41.06±1.77a	45.24±1.66a	4.51±0.09a	4.25±0.09b					
STIII	MP	35.26±1.22a	33.92±1.83a	4.54±0.07a	4.12±0.16b	Dullish	Grooved	Straight or inversely curved	Flexible	Terminal pores
	LP	33.23±0.95a	33.99±1.36a	4.94±0.09a	4.82±0.09a					
SCh	MP	146.78±8.48a	129.86±9.06 a	6.79±0.19a	6.10±0.14b	Sharp	Grooved	Curved	Flexible	No pore
	LP	151.91±6.07a	152.69±10.56a	7.43±0.18a	7.06±0.16a					
SP	MP	30.96±0.67a	30.74±0.56a	2.35±0.06b	2.72±0.04a	Flat	Smooth	Straight	Flexible	Terminal pore
	LP	28.12±0.40a	26.95±0.78a	2.57±0.07a	2.63±0.04a					
SCo	MP	2.06±0.05a	1.88±0.08a	1.68±0.03a	1.58±0.04a	Dullish	Smooth	Straight	Inflexible	Terminal pore
	LP	1.96±0.04a	2.00±0.06a	1.72±0.02a	1.55±0.04b					
CP	MP	—	—	0.58±0.02a	0.59±0.02a	—	—	—	—	—
	LP	—	—	0.61±0.03b	0.77±0.06a					
STBI	MP	5.33±0.09a	5.58±0.09a	2.27±0.03a	2.21±0.03a	Dullish	Smooth	Straight	Inflexible	Terminal pore
	LP	5.27±0.11b	6.05±0.10a	2.38±0.04a	2.33±0.03a					
STBII	MP	5.00±0.15a	5.11±0.14a	2.59±0.06a	2.36±0.04b	Blunt	Smooth	Straight	Inflexible	Terminal pore
	LP	5.42±0.12b	5.81±0.15a	2.63±0.04a	2.45±0.06b					
STBIII	MP	4.75±0.15a	5.03±0.17a	2.95±0.07a	2.77±0.05b	Blunt	Smooth	Straight	Inflexible	Terminal pore
	LP	4.87±0.08a	4.89±0.16a	3.00±0.03a	2.84±0.05b					
STBIV	MP	5.22±0.11a	5.42±0.09a	3.99±0.05a	3.97±0.04a	Blunt	Smooth	Straight	Inflexible	Terminal pore
	LP	5.44±0.08a	5.16±0.10b	4.05±0.03a	3.97±0.06a					

MP, maxillary palp; LP, labial palp. Data are presented as mean±SE. Within a row, different lowercase letters indicate a significant difference between the sexes (*t*-test, *P*<0.05). The basal diameter of the SP is the widest part.

**Table 8:** Abundance and distribution of different sensillum types/subtypes on the maxillary and labial palps of male and female *Mo. saltuarius*.

Organ	Segment	Sex	BB	CP	STII	STIII	SCh	SP	SCo	STBI	STBII	STBIII	STBIV	Total	Density
Maxillary palp	M1	♀	4.78±1.16a	0.89±0.42a	0.63±0.26a	—	0.33±0.17a	—	—	—	—	—	—	6.56±1.18a	0.20±0.04a
		♂	4.14±1.06a	1.14±0.34a	0.43±0.30a	—	0.00±0.00a	—	—	—	—	—	—	5.71±0.75a	0.13±0.02a
	M2	♀	—	15.78±2.41a	19.56±2.22a	4.11±0.90a	1.11±0.20a	—	—	—	—	—	—	40.56±4.35a	0.40±0.03a
		♂	—	14.71±2.97a	23.86±1.90a	3.57±0.43a	1.43±0.30a	—	—	—	—	—	—	43.57±3.89a	0.46±0.09a
	M3	♀	—	29.89±3.82a	20.44±4.44a	11.33±0.65a	1.11±0.20a	—	—	—	—	—	—	62.78±6.54a	0.89±0.10a
		♂	—	20.57±4.67a	17.00±1.68a	10.14±0.40a	1.00±0.22a	—	—	—	—	—	—	48.71±5.18a	0.64±0.08a
	M4	♀	—	117.33±9.11a	56.11±3.49a	15.89±0.95a	2.00±0.24a	13.22±3.70a	7.56±1.60a	20.00±2.62a	10.50±2.06a	8.67±0.84a	12.67±1.65a	246.67±15.01a	1.79±0.13a
		♂	—	132.00±5.89a	52.29±5.37a	12.29±0.84b	1.29±0.29a	14.14±3.88a	11.71±1.29a	27.83±3.20a	7.33±0.76a	7.83±1.08a	17.00±4.16a	275.14±12.95a	2.01±0.12a
	Total	♀	4.78±1.16a	163.89±12.94a	96.67±7.03a	31.33±1.42a	4.56±0.58a	13.22±3.70a	7.56±1.60a	20.00±2.62	10.50±2.06	8.67±0.84a	12.67±1.65a	392.00±14.96a	1.05±0.05a
		♂	4.14±1.06a	168.43±10.81a	93.57±7.11a	26.00±1.35b	3.71±0.61a	14.14±3.80a	11.71±1.29a	27.83±3.20	7.33±0.76	7.83±1.08a	17.00±4.16a	374.50±17.17a	1.07±0.08a
Labial palp	L1	♀	7.14±1.28a	2.43±0.20a	0.57±0.20a	—	0.57±0.30a	—	—	—	—	—	—	10.71±1.48a	0.37±0.05a
		♂	9.17±1.58a	2.67±1.02a	0.17±0.17a	—	0.50±0.22a	—	—	—	—	—	—	12.50±2.49a	0.38±0.08a
	L2	♀	—	25.71±2.07a	28.71±2.77b	17.86±1.55a	2.71±0.52a	—	—	—	—	—	—	75.00±3.72a	0.69±0.02a
		♂	—	20.50±3.08a	24.33±2.46a	17.00±1.13a	3.17±1.17a	—	—	—	—	—	—	65.00±5.05a	0.62±0.05a
	L3	♀	—	108.57±15.70a	53.43±3.73a	15.86±1.20a	2.14±0.40a	9.29±0.75a	11.86±1.47a	15.29±2.33a	18.71±1.64a	9.57±1.25a	16.00±2.39a	260.71±14.17a	1.74±0.15a
		♂	—	107.83±7.22a	61.00±7.54a	16.67±1.61a	1.17±0.40a	8.17±1.78a	9.17±1.62a	17.50±1.38a	9.33±1.20b	8.17±0.48a	13.83±3.19a	252.83±6.17a	1.71±0.06a
	Total	♀	7.14±1.28a	136.71±17.65a	82.71±4.74a	33.71±2.03a	5.43±0.69a	9.29±0.75a	11.86±1.47a	15.29±1.38a	18.71±1.64a	9.57±1.25a	16.00±2.39a	346.43±14.93a	1.21±0.08a
		♂	9.17±1.58a	131.00±10.04a	85.50±6.80a	33.67±1.54a	4.83±1.19a	8.17±1.78a	9.17±1.62a	17.50±1.64a	9.33±1.20b	8.17±0.48a	13.83±3.19a	330.33±9.80a	1.14±0.04a

Data are presented as the mean±SE. Within a column, different lowercase letters indicate significant differences between sexes (*t*-test, *P*<0.05). Density is expressed as the number per 10^3^ μm^2^. *n*=6 per sex. “—” indicates absence.

#### 3.3.2 STs

STIIs on the maxillary and labial palps were hair-like sensilla, with their bases located in flexible sockets; they were straight overall, with longitudinal grooves on the surface and sharp tips (**Figure 5B**). STIIs on female maxillary palps were 38.75±0.98 μm long (*n=*51) and were not significantly longer (*t*=−1.574, *df*=89, *P*=0.119) than those on male maxillary palps (41.85±1.84 μm, *n=*40). The basal diameter of STIIs on female maxillary palps was 3.99±0.08 μm (*n=*51), significantly smaller (*t*=−2.366, *df*=89, *P*=0.020) than that on male maxillary palps (4.29±0.09 μm, *n=*40). STIIs on female labial palps were 41.06±1.77 μm long (*n=*50) and were not significantly longer (*t*=−1.727, *df*=98, *P*=0.087) than those on male labial palps (45.24±1.66 μm, *n=*50). The basal diameter of STIIs on female labial palps was 4.51±0.09 μm (*n=*50), significantly larger (*t*=2.106, *df*=98, *P*=0.038) than that on male labial palps (4.25±0.09 μm, *n=*50). STIIIs were located within flexible sockets on the surface of the maxillary and labial palps. They were straight or inversely curved, with longitudinal grooves, and stood at a 60°–90° angle to the palp surface. One to several terminal pores were visible beneath their hat-like tip structures (**Figures 5C, D**). On female maxillary palps, STIIIs measured 35.26±1.22 μm in length (*n=*50), which was not significantly greater than in males (33.92±1.83 μm, *n=*21; *t*=0.603, *df*=69, *P*=0.548; **Table 7**). However, their basal diameter was significantly larger in females (4.54±0.07 μm, *n=*50) than in males (4.12±0.16 μm, *n=*21; *t*=2.847, *df*=69, *P*=0.006; **Table 7**). On the labial palps, STIIIs in females measured 33.23±0.95 μm in length and 4.94±0.09 μm in basal diameter (*n=*50), values that did not differ significantly from those in males (33.99±1.36 µm length, 4.82±0.09 μm diameter, *n=*32; *t_1_* =−0.471, *df_1_ =* 80, *P_1_ =* 0.639; *t_2_ =* 0.927, *df_2_ =* 80, *P_2_ =* 0.357; **Table 7**).

STIIs were widely distributed and abundant on the maxillary palps of both males and females, increasing in number from the base to the tip. The number of STIIs did not differ significantly between males and females. STIIs were also present on all segments of the labial palps in males and females, increasing in number from the base to the tip. The number of STIIs on L1 and L3, as well as the total number of STIIs on the labial palps, did not differ significantly between the sexes. However, there were significantly fewer STIIs on L2 in females than in males (**Table 8**). STIIIs were present on M2–M4 of both males and females, with their numbers increasing from the base to the tip. The total number of STIIIs, as well as the number on M4, was significantly greater in females than in males, whereas no sex-based differences were observed on M2 or M3. STIIIs were also detected on L2 and L3 in both sexes, with no significant differences in their numbers (**Table 8**).

#### 3.3.3 SChs

SChs were slender and elongated, with most curved, and a few standing upright. Each sensillum was set in a flexible socket, had longitudinal grooves along the surface, and ended in a sharp tip (**Figure 5B**). SChs on female maxillary palps were 146.78±8.48 μm long (*n=*21) and were not significantly longer (*t*=1.364, *df*=38, *P*=0.181) than those on male maxillary palps (129.86±9.06 μm, *n=*19). The basal diameter of SChs on female maxillary palps was 6.79±0.19 μm (*n=*21), significantly larger (*t*=2.910, *df*=38, *P*=0.006) than that on male maxillary palps (6.10±0.14 μm, *n=*19). SChs on female labial palps were 151.91±6.07 μm long (*n=*31) with a basal diameter of 7.43±0.18 μm (*n=*31); these values did not differ significantly (*t_1_* =−0.065, *df_1_ =* 59, *P_1_ =* 0.949; *t_2_ =* 1.554, *df_2_ =* 59, *P_2_ =* 0.126) from those of male labial palps, which were 152.69±10.56 μm long (*n=*30) with a basal diameter of 7.06±0.16 μm (*n=*30; **Table 7**).

On the maxillary palps of females, the number of SChs increased from the base to the tip; it was lowest on M1 (0.33±0.17) and highest on M4 (2.00±0.24). By contrast, on the maxillary palps of males, SCh number initially increased, then decreased from the base to the tip; no SChs were observed on M1, and the largest number were observed on M2 (1.43±0.30). SCh number did not differ between males and females for any segment of the maxillary palps. On both male and female labial palps, SCh number increased and then decreased from the base to the tip; it was highest on L2 for both females (2.71±0.52) and males (3.17±1.17). SCh numbers did not differ significantly between the sexes, either for individual segments or for the labial palps as a whole (**Table 8**).

#### 3.3.4 Sensilla placodea

SPs were flat with a blunt tip, a smooth surface, and a wide socket. Their upper surface was flush with the surface of the maxillary or labial palp, and they were separated from the organ’s surface on all sides (**Figures 5E, F**). SPs exhibited different widths at either end, with a pore present at the tip of the narrower end (**Figure 5G**). SPs on female maxillary palps were 30.96±0.67 μm long (*n=*18) and were not significantly longer (*t*=0.248, *df*=48, *P*=0.805) than those on males (30.74±0.56 μm). However, SPs on maxillary palps were significantly narrower in females (2.35±0.06 μm, *n=*18) (*t*=−5.087, *df*=48, *P*=0.000) than in males (2.72±0.04 μm, *n=*32). By contrast, there were no significant differences (*t_1_ =* 1.463, *df_1_ =* 62, *P_1_ =* 0.149; *t_2_* =−0.842, *df_2_ =* 45, *P_2_ =* 0.404) in the length or width of SPs on the labial palps of females versus males: those of females were 28.12±0.40 μm long (*n=*39) and 2.57±0.07 μm wide (*n*=22), and those of males were 26.95±0.78 μm long (*n=*25) and 2.63±0.04 μm wide (*n*=25).

SPs were found exclusively on M4 in both males and females, and SP number did not differ significantly between the sexes. Likewise, SPs were found exclusively on L3 in both males and females, and their numbers did not differ between the sexes (**Table 8**).

#### 3.3.5 Sensilla coeloconica

SCos were conical in shape, situated within a distinct concavity on the basal socket, with a smooth surface and a terminal pore (**Figures 5H, I**). SCos on female maxillary palps were 2.06±0.05 μm long (*n=*33) with a basal diameter of 1.68±0.03 μm (*n=*33); they did not differ significantly (*t_1_ =* 1.961, *df_1_ =* 46, *P*=0.056; *t_2_ =* 1.616, *df_2_ =* 46, *P*=0.113) from SCos on male maxillary palps, which were 1.88±0.08 μm long (*n=*15) with a basal diameter of 1.58±0.04 μm (*n=*15). SCos on female labial palps were 1.96±0.04 μm long (*n=*36) and did not differ significantly in length (*t*=−0.606, *df*=53, *P*=0.547) from those on male labial palps (2.00±0.06 μm, *n=*19). The basal diameter of SCos on female labial palps was 1.72±0.02 μm (*n=*36), significantly larger than that on male labial palps (1.55±0.04 μm, *n=*19; **Table 7**). SCos were present on M4 and L3 in both males and females, and their numbers did not differ between the sexes (**Table 8**).

#### 3.3.6 CPs

CPs appeared as small pores on the surface of the maxillary and labial palps (**Figure 5H**). CP diameter on female maxillary palps was 0.58±0.02 μm (*n=*50) and did not differ significantly from that on male maxillary palps (0.59±0.02 μm, *n=*52) (*t*=0.422, *df*=100, *P*=0.674). CP diameter on female labial palps was 0.61±0.03 μm (*n=*56), significantly smaller (*t*=−2.671, *df*=104, *P*=0.009) than that on male labial palps (0.77±0.06 μm, *n=*50; **Table 7**). CPs were found across all segments of the maxillary palps in both males and females, gradually increasing in number from the base to the tip. CP number did not differ significantly between males and females on any maxillary palp segments. CPs were also found across all segments of the labial palps in both males and females, increasing gradually in number from the base to the tip. Neither the number of CPs in individual segments nor the total number of CPs differed significantly between males and females (**Table 8**).

#### 3.3.7 Sensilla twig basiconica

**Figure 6:**
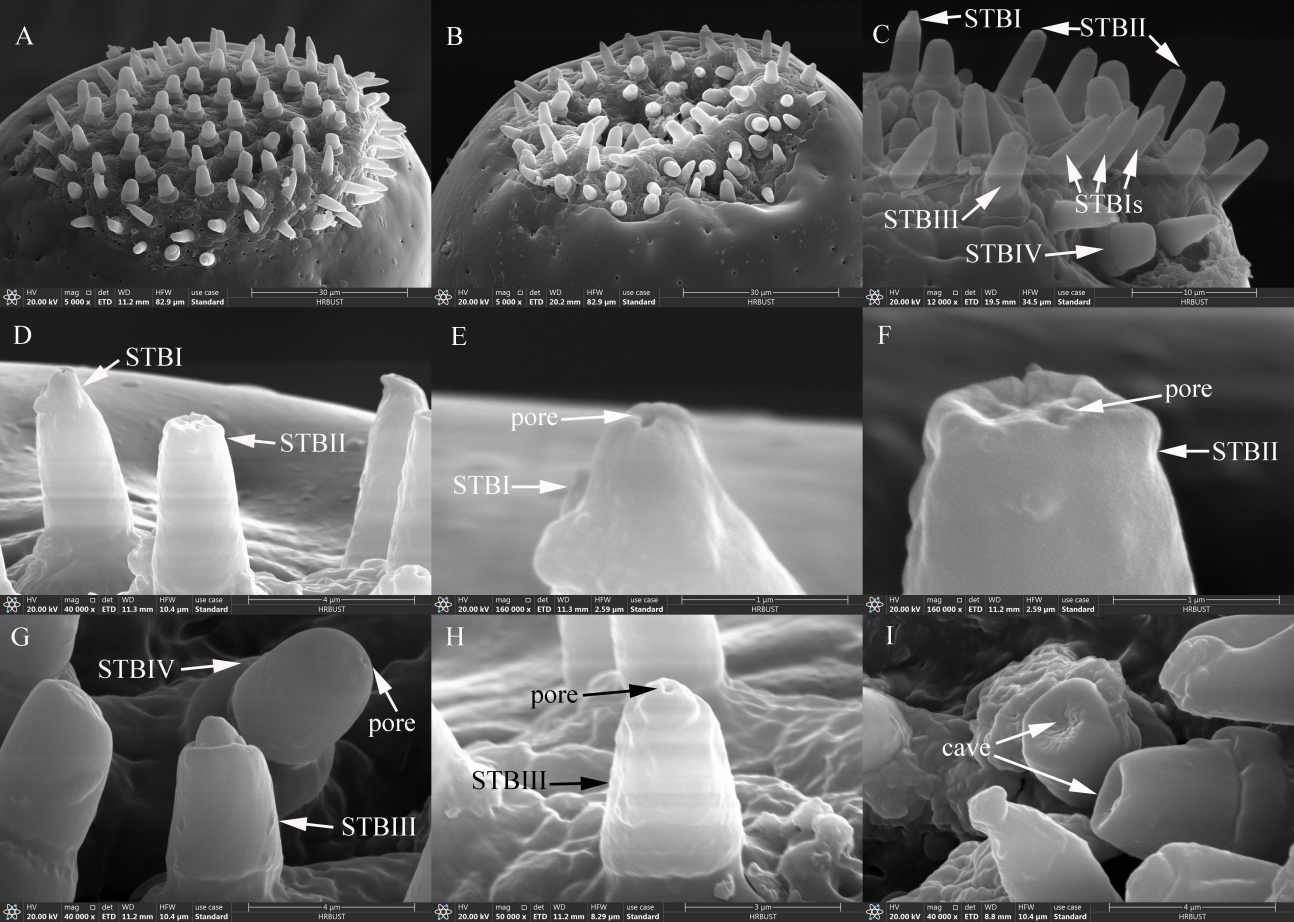
Ultrastructure of STBs on maxillary and labial palps of adult *Mo. saltuarius*. **(A)** Sensilla on the maxillary palp tip; **(B)** sensilla on the tip of the labial palp; **(C)** ultrastructure of four STB subtypes; **(D)** STBI and STBII; **(E)** terminal pore of an STBI; **(F)** terminal pores of an STBII; **(G)** STBIII and STBIV; **(H)** ultrastructure of an STBIII; **(I)** terminal cave of an STBIV.

STBs on the maxillary palps were densely distributed at the tip of M4 (**Figure 6A**), whereas those on the labial palps were densely distributed at the tip of L3 (**Figure 6B**). STBs on the maxillary and labial palps consisted of four subtypes: STBIs, STBIIs, STBIIIs, and STBIVs. STBIs exhibited a conical shape; they were straight, with an inflexible cuticular socket, a smooth wall, and a smaller finger-like projecting tip with a pore (**Figures 6C–E**). STBIs on female maxillary palps were 5.33±0.09 μm long with a basal diameter of 2.27±0.03 μm; they did not differ significantly from those on male maxillary palps, which were 5.58±0.09 μm long with a basal diameter of 2.21±0.03 μm (**Table 7**). STBIs were significantly shorter on female labial palps (5.27±0.11 μm) than on male labial palps (6.05±0.10 μm), but they did not differ in basal diameter between females (2.38±0.04 μm) and males (2.33±0.03 μm; **Table 7**). The number of STBIs on female maxillary palps was 20.00±2.62 and did not differ significantly from that on male maxillary palps (27.83±3.20; **Table 8**). Likewise, the number of STBIs on female labial palps (15.29±1.38) did not differ significantly from that on male labial palps (17.50±1.64; **Table 8**).

STBIIs exhibited a conical shape with an inflexible cuticular socket and a smooth wall; they had a blunt, rounded tip with a central radial groove and one to several terminal pores (**Figures 6C–D, F**). STBIIs on female maxillary palps were 5.00±0.15 μm long and did not differ significantly in length from those on male maxillary palps (5.11±0.14 μm). However, the basal diameter of STBIIs was significantly greater on maxillary palps of females (2.59±0.06 μm) than of males (2.36±0.04 μm; **Table 7**). STBIIs on female labial palps were 5.42±0.12 μm long, significantly shorter than those on males (5.81±0.15 μm), whereas STBII basal diameter was significantly larger on female (2.63±0.04 μm) than on male labial palps (2.45±0.06 μm; **Table 7**). The number of STBIIs on maxillary palps did not differ between females (10.50±2.06) and males (7.33±0.76; **Table 8**), but the number of STBIIs on labial palps was significantly higher in females (18.71±1.64) than in males (9.33±1.20; **Table 8**).

STBIIIs exhibited a two-tiered morphology; the lower tier was robust and cylindrical, whereas the upper tier had a smaller conical shape with a distinct terminal pore (**Figures 6C**, G–H). The length of STBIIIs on the maxillary palps did not differ between females (4.75±0.15 μm) and males (5.03±0.17 μm; **Table 7**), but their basal diameter was significantly larger in females (2.95±0.07 μm) than in males (2.77±0.05 μm; **Table 7**). Likewise, the length of STBIIIs on the labial palps did not differ significantly between females (4.87±0.08 μm) and males (4.89±0.16 μm; **Table 7**), but their basal diameter was significantly greater in females (3.00±0.03 μm) than in males (2.84±0.05 μm; **Table 7**). There were also no differences between the sexes in STBIII numbers on the maxillary palps (8.67±0.84 in females, 7.83±1.08 in males; **Table 8**) or labial palps (9.57±1.25 in females, 8.17±0.48 in males; **Table 8**).

STBIVs had a wide socket, a blunt tip, and smooth lateral walls; an apical pore or cave was visible (**Figures 6G, I**). However, a longitudinal section of a similar sensillum shaft in *Xylotrechus grayii* showed micro apical pores on the tip (25), and the presence of such pores cannot be ruled out in *Mo. saltuarius*. STBIVs were commonly located in a central position on the tips of maxillary and labial palps (**Figures 6A, B**). On female maxillary palps, STBIVs were 5.22±0.11 μm long with a basal diameter of 3.99±0.05 μm; these measurements did not differ significantly from those in males (length 5.42±0.09 μm; basal diameter 3.97±0.04 μm; **Table 7**). STBIVs on female labial palps were 5.44±0.08 μm long, significantly longer than those of males (5.16±0.10 μm). The basal diameter of female labial palps was 4.05±0.03 μm and did not differ significantly from that of males (3.97±0.06 μm; **Table 7**). Numbers of STBIVs on the maxillary and labial palps did not differ between females (12.67±1.65 and 16.00±2.39) and males (17.00±4.16 and 13.83±3.19; **Table 8**).

#### 3.3.8 Distribution of sensilla on the maxillary palps

The density and number of sensilla increased gradually from the base to the tip of the maxillary palps in both females and males. The total number and density of sensilla on M1–M4 did not differ significantly between the sexes (**Table 8**). M1 segments of females contained 4 sensillum types/subtypes: BBs (72.87%), CPs (13.57%), STIIs (9.60%), and SChs (5.03%). By contrast, M1 segments of males contained only 3 sensillum types/subtypes: BBs (72.50%), CPs (19.96%), and STIIs (7.53%). M2 segments of males and females contained four sensillum types/subtypes: STIIs (48.22% in females and 54.75% in males), CPs (38.90% and 33.77%), STIIIs (10.14% and 8.20%) and SChs (2.74% and 3.28%). M3 segments contained the same four sensillum types/subtypes as M2s in both sexes: CPs (47.61% in females and 42.23% in males), STIIs (32.57% and 34.90%), STIIIs (18.05% and 20.82%), and SChs (1.77% and 2.05%). M4 segments contained 10 sensillum types/subtypes in both females and males; in addition to those found on the M3s, M4s also contained SCos, SPs, STBIs, STBIIs, STBIIIs, and STBIVs.

#### 3.3.9 Distribution of sensilla on the labial palps

The total number and density of sensilla on the labial palps increased gradually from the base to the tip in both males and females (**Table 8**), and there were no significant differences between the sexes in the number or density of sensilla on corresponding segments. L1 segments contained four sensillum types/subtypes in both sexes: BBs (66.67% in females and 73.33% in males), CPs (22.67% and 21.33%), STIIs (5.33% and 1.33%), and SChs (5.33% and 4.00%). L2 segments also contained four sensillum types/subtypes in both sexes: STIIs (38.29% in females and 37.44% in males), CPs (34.29% and 31.54%), STIIIs (23.81% and 26.15%), and SChs (3.62% and 4.87%). L3 segments contained 10 sensillum types/subtypes in both sexes; in addition to those found on the L2s, L3s also contained SCos, SPs, STBIs, STBIIs, STBIIIs, and STBIVs.

## 4 Discussion

Insect sensilla are classified on the basis of multiple criteria: the shapes of outer cuticular structures such as hairs and plates, the thickness of the cuticular wall, the flexibility of the cuticular socket, the presence and types of cuticular pores, the dendritic branching patterns, and the numbers of innervating sensory neurons (21). Sensilla that function as mechanoreceptors typically lack pores, as do thermo- or hygrosensilla (3, 21, 28). The presence of pores typically indicates that a sensillum functions in chemoperception. A single apical pore is characteristic of gustatory sensilla, whereas multiple pores are typically present on olfactory sensilla (22), a difference that likely reflects the different functions of these sensillum types: olfactory sensilla detect pheromones and plant volatiles at a distance (3, 29, 30), whereas gustatory sensilla detect non-volatile phytochemicals and pheromones through direct contact (3, 31, 32).

The antennae, maxillary palps, labial palps, and associated sensilla of insects are thought to reflect long-term adaptation to specific environments (33, 34). The presence of similar sensillum types in insects also indicates that they are evolutionarily related (26, 35, 36). We can therefore refer to information on other longhorn beetles from the same family or subfamily to gain insight into the types and functions of sensilla in *Mo. saltuarius*. Longhorn beetles of the Cerambycidae family have up to 13 types of antennal sensilla (24 subtypes), all of which are present in the Cerambycinae subfamily (37–41). By contrast, the Lamiinae subfamily has 9 types (16 subtypes) (42–44), the Lepturinae subfamily has 6 types (9 subtypes) (45), and the Aseminae subfamily has 6 types (10 subtypes) (46).

The Cerambycidae genera *Xylotrechus* and *Massicus* exhibit the highest diversity of antennal sensilla, with each genus possessing over 20 distinct subtypes (37, 47). Although the types of antennal sensilla differ among genera and species, some (such as STs, SChs, SBs, SAus, SGPs, DSOs, and BBs) are found in most longhorn beetles (48, 49), and STs, SChs, and SBs are present in nearly all longhorn beetle species (43). *Xylotrechus* and *Massicus* (Cerambycinae) (37, 47) and *Saperda* and *Monochamus* (Lamiinae) (20, 42, 49) have SAus, SGPs, and DSOs, but none of these sensilla are found in *Nadezhdiella* and *Phoracantha* (Cerambycidae) or *Coscinesthes* (Lamiinae). SGP and DSO sensilla are found in both Lepturinae and Aseminae, but SAus are not present in either subfamily (45, 46).

BBs function in proprioception and are found on all arthropod antennae (3, 4). Here, BBs were observed on the antennae, maxillary palps, and labial palps of *Mo. saltuarius* and were similar to BBs described in *Glenea cantor* (50), *Aromia bungii* (40), *Xylotrechus grayii* (51), *Xylotrechus quadripes* (26), and *Pharsalia antennata* (52). BBs are widely considered to function as mechanoreceptors for gravity perception. Mechanoreceptors on the antennae respond to touch, vibration, and airflow by striking the surface of the antennal flagellum ventrally. BBs occur at the radicle, at the scape–pedicel junction, and at the bases of M1 and L1, consistent with a role in the perception of antennal position and movement (53–55); these locations may provide an optimal angle for sensing antennal position and obtaining precise positioning signals (56).

STIs were most numerous and widely distributed on the antennae of *Mo. saltuarius*, and their densities on antennae were higher in females than in males. The distribution of *Mo. saltuarius* STIs was similar to that of sensilla described previously under various names: SBLIs in *Monochamus alternatus* (23), SC1s in *X. quadripes* (26), ST-IVs and ST-Vs in *Pharsalia antennata* (52), STIIs in *Rhaphuma horsfieldi* (41), and stout SCs in *Monochamus notatus*, *Monochamus scutellatus*, and *Monochamus galloprovincialis* (20, 57). Gland openings or clusters of pores immediately adjacent to STI insertions have been reported previously (20, 57). Because STIs lack pores and show limited innervation, they are unlikely to function in chemoperception (20, 46, 58). Álvarez et al. (57) were unable to record action potentials from STIs (which they termed stout SCs), suggesting that they may be mechanoreceptors. Although STIs are frequently classified as a single subtype, some species have multiple STI subtypes that differ in size and morphology, depending on their antennal distribution (38, 51, 57). STIs on the antennae of male *Mo. saltuarius* are similar to the ‘bottle-like sensilla’ described in *Mo. alternatus* (59) and the ‘male peg SCs’ described in *Mo. galloprovincialis* (57).

The distribution of *Mo. saltuarius* STIIs was similar to that of sensilla described in other species under a variety of names: SC3s in *X. quadripes* (26), STIs in *Pha. antennata* (52), and STIs in *R. horsfieldi* (41). STIIs also have the general characteristics of a mechanoreceptor.

The morphology of STIIIs in *Mo. saltuarius* resembled that of SRLs in *Mo. alternatus* (23), STr2s in *Mo. alternatus* and *Mo. saltuarius* (19), STs in *Mo. notatus* and *Mo. scutellatus* (20), ST1s and ST2s in *Chlorophorus caragana* (60), uniporous sensillum chaeticum in *Phoracantha recurva* (38), finger sensilla 2 in *X. rusticus* (37), ST1s in *X. quadripes* (26), and ST2s in *Allotraeus asiaticus* (61). STIIIs of *Mo. saltuarius* exhibited longitudinal grooves on their surfaces and one to several terminal pores, classifying them as groove-type chemoreceptors. This sensillum subtype contains an apical pore and is innervated by an average of 7–8 neurons/hair in *Mo. notatus* and *Mo. scutellatus*, suggesting a role in contact chemoperception. Here, STIIIs were found on the antennae as well as the maxillary and labial palps of adult *Mo. saltuarius*. We infer that STIIIs likely serve as the main contact chemoreceptors, with functions in both mechanical and chemical stimulus perception.

SChI morphology in *Mo. saltuarius* resembled that of sensilla described previously in *G. cantor* (50). These sensilla have been termed SC1s in *Tetropium fuscum* (46), SC2s in *X. grayii* (51), SChIVs in *Pha. antennata* (52), SChIIs in *R. horsfieldi* (41), SCIs in *X. quadripes* (26), and long SCs in *Mo. notatus*, *Mo. scutellatus*, and *Mo. galloprovincialis* (20, 57). Transmission electron microscopy observations of SChIs in *Mo. notatus* and *Mo. scutellatus* revealed that these sensilla are innervated by a single sensory neuron (20). In Hymenopteran wasps, SChs serve as tactile mechanoreceptors that sense relative antennal position (62), and some studies have suggested that SChs are involved in host exploration and identification (63, 64). The SChIs on the antennae of *Mo. saltuarius* are oriented almost perpendicularly to the antennal surface and are much longer than other sensilla, enabling them to make contact with objects first and suggesting that they are also mechanoreceptors.

The morphology of SChIIs resembled that of SCs in *Massicus raddei* (39), SChIIIs in *G. cantor* (50), SSTIs in *Mo. alternatus* (23), SC4s in *X. quadripes* (26), ChIs in *Plagionotus pulcher* (65), SChIIIs in *Pha. antennata* (52), SChIs in *R. horsfieldi* (41), and distal SCs in *Mo. notatus* and *Mo. scutellatus*, as well as large SCs in *Mo. galloprovincialis* (57). SChIIs are aporous and much longer than other nearby sensillum types. As with other longhorn beetles, *Mo. saltuarius* had SChIIs at junctions where one flagellomere overlapped with the proximal portion of the next flagellomere (3). SChIIs, like SChIs, are innervated by one sensory neuron (20), and research in *Mo. galloprovincialis* demonstrated that their neurons produced action potentials in response to movement (57). These characteristics suggest that SChIIs are proprioceptors that detect the positioning of antennal segments.

The SAu of *Mo. saltuarius* was a spoon- or ear-shaped structure with a relatively flat or concave distal surface and was found mainly on the antennal flagellum. The morphology of SAus resembled that of SBIIs in *G. cantor* (50), SBIIIs in *Mo. alternatus* (23), SAus in *Mo. alternatus* (58), stout SBs in *Mo. notatus* (20), SB3s in *X. grayii* (51), SBIs in *Leptura arcuata* (45), Aus in *Ch. caragana* (60), SAs in *X. quadripes* (26), SBIs in *Pha. antennata* (52), and SBIs and SBIIs in *R. horsfieldi* (41). SAus in *Mo. alternatus* had thin walls, abundant cuticular pores, and fewer than five dendritic branches (58). These features suggest that they have an olfactory function, and many studies have proposed that this sensillum may sense the stimulation of host volatiles (54, 66).

The morphology of SBIs in *Mo. saltuarius* resembled that of SBIs in *G. cantor* (50), STs in *Mo. alternatus* (58), SBIIs in *Pho. recurva* (38), SBIIs in *L. arcuata* (45), SBIIs in *Leptura aethiops* (45), SB2s in *X. quadripes* (26), B1s in *Pl. pulcher* (65), SBIIs in *Pha. antennata* (52), and SB1s in *R. horsfieldi* (41). SBIs in *Mo. alternatus* (their ‘sensilla trichodea’) had somewhat thicker walls with numerous pores (but fewer than SAus) and were innervated by 1–8 dendrites (58); these SB subtypes also had multiporous walls and were innervated by multiple dendritic branches in *T. fuscum*, indicative of an olfactory function (46). SBIIs in *Mo. saltuarius* resembled SB1s in *X. grayii* (51), SBIVs in *L. arcuata* and *L. aethiops* (45 SC2s in *Mo. alternatus* (58), and SBIIIs in *Pha. antennata* (52). SBIIs are generally believed to be taste chemoreceptors used for host recognition (21, 67, 68), and taste function (63, 64, 69).

SBIs and SBIIs in *Mo. saltuarius* were found mainly on the antennal flagellum; they increased gradually in number from the base to the tip and were often found in dense patches on each flagellomere (**Figure 4C**). The concentration of SBs (**Figure 4D** and the dashed-line area in **Figure 4C**) and the differences in their numbers between males and females (both SBIs and SBIIs were higher in males than in females, **Table 6**) are shown in *Mo. saltuarius*. This distribution pattern—characterized by a dense sensory region— is similar to that of SBs in *Pl. pulcher* (65), *Mo. notatus*, and *Mo. scutellatus* (20) and is believed to be beneficial for the detection of odor molecules and the perception of sexual information released by conspecifics in *X. grayii* (51), *X. rusticus* (37), *Phoracantha semipunctata* (38), and *Pho. recurva* (38). Different SB subtypes may selectively recognize external chemical information (37, 38).

The morphology of SGPs resembled that of grooved peg sensilla in *Aedes aegypti* (70), *X. grayii* (51) and *X. quadripes* (26), SGPs in *X. rusticus* (37) and *T. fuscum* (46), Stys in *Mo. alternatus* (58) and *Ch. caragana* (60), SBIIIs in *L. arcuata* (45) and *L. aethiops* (45), and B6s in *Pl. pulcher* (65). Pores on the proximal smooth cuticle were few and shallow, and no pores were present on the grooved portion of the sensillum (58, 60). However, pore channels run along the grooves of these sensilla in *A. aegypti* with approximately 38 pore openings per groove, totaling about 456 openings per peg (70). In *Pho. semipunctata*, each sensillum is innervated by 4–5 bipolar neurons (71). Based on their ultrastructure, SGPs are generally thought to possibly have a dual role as both thermal and chemical sensilla (21, 63, 70, 72).

The morphology of DSOs resembled that of SCas in *Callidiellum villosulum* (61), Cas in *X. quadripes* (26), dome organs (campaniform) in *Mo. scutellatus* (20), SCos in *Mo. alternatus* and *Mo. saltuarius* (19), SCs in *Pl. pulcher* (65), and SCas in *Pha. antennata* (52). These sensilla in *Mo. scutellatus* are innervated by at least one neuron per sensillum (20). Electrophysiological evidence in *Pterostichus aethiops* (73) and *Pt. oblongopunctatus* (74) suggests that they are thermo- and hygroreceptors.

The morphology of CPs resembled that of CPs in *X. quadripes* (26) and *X. rusticus* (37) and of the ms in *Ma. raddei* (39). CPs have been described as similar sensory organs in various families of Coleoptera, including Carabidae, Chrysomelidae, Meloidae, Paussidae, Pselaphidae, Silphidae, and Staphylinidae. CPs were once thought to be pheromone glands, kairomones, or lubricants for antennae and sensilla (75). In *Coccinella septempunctata*, CPs were described as sensilla ampucellaceous (Ams) with potential chemical and/or thermal sensory functions (76). In moth antennae, CPs may have enzymatic functions that prevent the accumulation of non-active pheromones and plant volatiles (77).

The morphology of SPs resembled that of sensilla placodea in *Ca. villosulum* (27), digitiform sensilla in *Mo. alternatus* (78) and *Ch. caragana* (24), and sensilla digitiformia in *X. grayii* (25). SPs were not found on the antennae of *Mo. saltuarius* but were present on the dorsal tip of the last segment of the maxillary (M4) and labial palps (L3). There was a pore at the top of one side of the SP, which is usually considered to function in chemoreception and mechanoreception or to sense changes in CO_2_, temperature, and humidity (79–81). The SPs of Coleoptera sense contact-vibration stimuli that may be related to the movement of insects in tunnels (82). The distribution of SPs on adult *Mo. saltuarius* at the dorsal distal segments of the maxillary and labialpalps may enable them to contact the inner wall of the tunnel, thus sensing the temperature, humidity, and vibrations caused by feeding of other similar insects. This enables them to adjust their feeding path and avoid tunnel overlap, and it may also help them to search for the most suitable feeding sites and summering places.

The morphology of SCos on the maxillary and labial palps of *Mo. saltuarius* resembled that of sensilla pit basiconica in *Anoplophora glabripennis* and *Anoplophora chinensis* (43), S.tb.5s in *Ch. caragana* (24), SCas in *Ca. villosulum* (27), short sensilla styloconica in *Philus antennatus* (83), and recessed peg sensilla in *Cicindela sexguttata* (Cicindelidae). Faucheux considered that their main function was as sensors for CO_2_, temperature, and humidity (84).

The morphology of STBIs on the maxillary and labial palps of *Mo. saltuarius* resembled that of Stb1s in *Mo. alternatus* (78), S.tb.3s in *Ch. caragana* (24), Sty2s in *X. grayii* (25), s.b.1s in *Siagona europaea* (85), and SBIVs in *Ca. villosulum* (27). These sensilla are innervated by 2–6 dendrites in *X. grayii* and have a thick dendritic sheath surrounded by tormogen and trichogen cells below the cuticle level (25). STBIs resemble uniporous sensilla that perceive odors through taste or contact (22, 86) and may function as contact chemoreceptors, detecting mechanical and chemical stimuli while performing gustatory and olfactory roles.

The morphology of STBIIs on the maxillary and labial palps of *Mo. saltuarius* resembled that of SBIIIs in *Ca. villosulum* (27), Stb2s in *Mo. alternatus* (78), S.tb.2s in *Ch. caragana* (24), S.t.b.2s in larval *An. glabripennis* (87), and S2s in larval *Melolontha melolontha* (88). These sensilla were thought to be contact gustatory sensilla in *Mo. alternatus* and larval *Me. melolontha* (78, 88).

The morphology of STBIIIs on the maxillary and labial palps of *Mo. saltuarius* resembled that of Stb3s in *Mo. alternatus* (78), S.tb.4s in the labial palps of *Ch. caragana* (24), Sty4s in *X. grayii* (25), S.t.b.6s in larval *An. glabripennis* (87), and sensilla styloconica in larval *An. glabripennis* (89). In *X. grayii*, these sensilla have 3 dendrites and are thought to sense chemical stimuli (25); they have also been identified as chemosensilla in larval *An. glabripennis* (87) and proposed to function as chemo-, thermo-, and hygroreceptors (25, 87, 90).

The morphology of STBIVs on the maxillary and labial palps of *Mo. saltuarius* resembled that of Stb4s in *Mo. alternatus* (78) and Sty3s in *X. grayii* (25). These sensilla are innervated by 3 dendrites surrounded by tormogen and trichogen cells in *X. grayii*; the dendrites are suspended under a thin dendritic sheath in the inner lymphatic cavity and extend into the shaft lumen (25). Sty3s in *X. grayii* have thin walls and small apical pores, which are typical of olfactory and taste sensilla (25). The STBIVs in *Mo. saltuarius* exhibited a cave on the tip surface, suggesting that the tip wall may be soft and thin; this structure probably contributes to the diffusion of odorants into the shaft. The location of the micro-apical pores may also indicate a gustatory function.

STBs were located on the apices of the maxillary and labial palps of adult *Mo. saltuarius* and may therefore play a role in adult feeding habits. They may help adults select good, fresh, and nourishing foods while avoiding harmful substances and may also be involved in detecting host-tree chemical cues, monitoring food texture, and evaluating food quality.

The STBs on the tips of the maxillary and labial palps are highly sensitive, because the olfactory sensillum dendrites are divided into many branches and the tips of the conical sensilla have many small pores that admit gas molecules and accept more molecules diffused from the host (91). The olfactory sensilla of both maxillary and labial palps can accept molecular stimuli that have diffused into the air from the host and do not require host contact, enabling the insects to detect their host tree; thus, these sensilla play a role in long-distance host selection. By contrast, taste sensilla can only confirm the presence of the host upon direct contact and are stimulated by dissolved molecules (91, 92).

The antennal composition and flagellomere number of male and female *Mo. saltuarius* were the same as those of *X. rusticus* (37), *Ar. bungii* (40), *G. cantor* (50), and *R. horsfieldi* (41). There were no significant differences between males and females in the density of olfactory sensilla on the antennae. However, antennal length and surface area were slightly greater in males than in females. Therefore, the number of chemoreceptors (STIIIs, SAus, SBIs, SBIIs, SGPs and CPs) and the total number of sensilla on the antennae were significantly greater in males (**Table 6**). There were no significant differences between males and females in the number of sensilla on the maxillary and labial palps.

## 5 Conclusion

We observed sexual dimorphism in the number, distribution, and morphology of sensilla on the antennae, maxillary palps, and labial palps of adult *Mo. saltuarius*. Both sexes contained the same sensillum types, which included putative chemoreceptors and/or mechanoreceptors. There were 8 types on the antennae and 7 types on the maxillary and labial palps. The antennal BBs, STIs, STIIs, SChIs, and SChIIs and the labial BBs, STIIs, and SChs may function as mechanoreceptors for proprioception, tactile perception, air-movement detection, and host location and exploration. The antennal SAus, SBIs, SBIIs, and SGPs may be olfactory chemoreceptors used during host searching, or mating. The STIIIs, SPs, STBIs, STBIIs, STBIIIs and STBIVs may be taste chemoreceptors used during feeding. The DSOs, and SCos may be receptors for water, and temperature. The types and densities of sensilla on the antennae of adult *Mo. saltuarius* increased from the base to the tip, and sensilla with chemical-sensing functions were concentrated mainly on the flagellum. By characterizing the morphology, number, and distribution of different sensillum types, we can better understand the olfactory receptive mechanisms that enable intraspecific and interspecific chemical communication in *Mo. saltuarius*. In future work, we hope to identify genes related to olfactory sensilla and characterize their roles in host location, mating, oviposition, and other processes in order to limit the damage caused by *Mo. saltuarius* through genetic manipulation.

## Data Availability

The raw data supporting the conclusions of this article will be made available by the authors, without undue reservation.
